# Engineering of Aromatic Naphthalene and Solvent Molecules to Optimize Chemical Prelithiation for Lithium‐Ion Batteries

**DOI:** 10.1002/advs.202309155

**Published:** 2024-06-18

**Authors:** Jagabandhu Patra, Shi‐Xian Lu, Jui‐Cheng Kao, Bing‐Ruei Yu, Yu‐Ting Chen, Yu‐Sheng Su, Tzi‐Yi Wu, Dominic Bresser, Chien‐Te Hsieh, Yu‐Chieh Lo, Jeng‐Kuei Chang

**Affiliations:** ^1^ Department of Materials Science and Engineering National Yang Ming Chiao Tung University 1001 University Road Hsinchu 30010 Taiwan; ^2^ Hierarchical Green‐Energy Materials (Hi‐GEM) Research Center National Cheng Kung University 1 University Road Tainan 70101 Taiwan; ^3^ International College of Semiconductor Technology National Yang Ming Chiao Tung University 1001 University Road Hsinchu 30010 Taiwan; ^4^ Department of Chemical Engineering and Materials Engineering National Yunlin University of Science and Technology 123 University Road Yunlin 64002 Taiwan; ^5^ Helmholtz Institute Ulm (HIU) Helmholtzstrasse 11 89081 Ulm Germany; ^6^ Karlsruhe Institute of Technology (KIT) 76021 Karlsruhe Germany; ^7^ Department of Chemical Engineering and Materials Science Yuan Ze University 135 Yuandong Road Taoyuan 32003 Taiwan; ^8^ Department of Chemical Engineering Chung Yuan Christian University 200 Chung Pei Road Taoyuan 32023 Taiwan

**Keywords:** density functional theory, hard carbon, methyl‐naphthalene, solution aging time, solvent selection

## Abstract

A cost‐effective chemical prelithiation solution, which consists of Li^+^, polyaromatic hydrocarbon (PAH), and solvent, is developed for a model hard carbon (HC) electrode. Naphthalene and methyl‐substituted naphthalene PAHs, namely 2‐methylnaphthalene and 1‐methylnaphthalene, are first compared. Grafting an electron‐donating methyl group onto the benzene ring can decrease electron affinity and thus reduce the redox potential, which is validated by density functional theory calculations. Ethylene glycol dimethyl ether (G1), diethylene glycol dimethyl ether, and triethylene glycol dimethyl ether solvents are then compared. The G1 solution has the highest conductivity and least steric hindrance, and thus the 1‐methylnaphthalene/G1 solution shows superior prelithiation capability. In addition, the effects of the interaction time between Li^+^ and 1‐methylnaphthalene in G1 solvent on the electrochemical properties of a prelithiated HC electrode are investigated. Nuclear magnetic resonance data confirm that 10‐h aging is needed to achieve a stable solution coordination state and thus optimal prelithiation efficacy. It is also found that appropriate prelithiation creates a more Li^+^‐conducing and robust solid‐electrolyte interphase, improving the rate capability and cycling stability of the HC electrode.

## Graphical abstract

This study optimizes polyaromatic hydrocarbons and solvents to develop cost‐effective and scalable chemical prelithiation recipes. The effects of the interaction time between Li metal, polyaromatic hydrocarbons, and solvent on prelithiation power are systematically investigated. An optimal recipe leads to a solid‐electrolyte interphase with a balanced organic and inorganic composition, resulting in enhanced initial Coulombic efficiency, rate capability, and cycling stability of the prelithiated electrode.

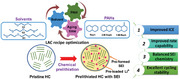

## 1 Introduction

Lithium‐ion batteries (LIBs) have become increasingly crucial in a wide range of applications, including portable electronics, electric vehicles, and grid‐scale energy storage systems, owing to their cost‐effectiveness, versatility, and reliability.^[^ ^1^, ^2^ ^]^ A higher energy density is a key target of LIB development for further market acceptance.^[^ ^3^, ^4^ ^]^ In this context, researchers have pursued high‐potential positive electrodes and low‐potential negative electrodes with high capacities.^[^ ^5^ ^]^ The amount of cyclable Li^+^ between positive and negative electrodes determines the reversible capacity of an LIB.^[^ ^6^ ^]^ Active Li^+^ loss due to factors such as the trapping of Li^+^ ions within the electrode lattice, solid‐electrolyte interphase (SEI) evolution, electrolyte decomposition, and dead Li formation can decrease reversible capacity. Because conventional graphite anodes have a limited capacity (≈350 mAh g^−1^), the development of advanced high‐capacity anodes is important.^[^ ^7^ ^]^ However, alternative anodes, such as hard carbon (HC), Si‐based, Sn‐based, and P‐based electrodes, usually suffer from significant Li^+^ loss in the first and subsequent charge‐discharge cycles.^[^ ^8^, ^9^ ^]^ This continuously consumes a considerable number of active Li^+^ ions, resulting in a substantial deterioration in the LIB energy density and cycle life.^[^ ^10^, ^11^ ^]^ Increasing the number of cyclable Li^+^ ions and reducing Li^+^ loss are thus worthy of further investigation.

Prelithiation has been proven to be a highly effective method for compensating for Li^+^ loss.^[^ ^6^, ^7^, ^8^, ^9^, ^10^, ^11^ ^]^ With prelithiation, Li^+^ ions are pre‐loaded into the electrodes before cell assembly. This Li^+^ inventory helps maintain the amount of cyclable Li^+^ in the battery.^[^ ^12^, ^13^, ^14^ ^]^ There are several prelithiation methods, including direct contact with Li metal, the use of stabilized lithium metal powder, the incorporation of prelithiation additives, electrochemical prelithiation, and chemical prelithiation.^[^ ^6^, ^8^, ^12^, ^14^ ^]^ The use of Li foil and powder poses some safety risks and usually results in low prelithiation uniformity. Prelithiation additives have limited efficacy and may leave behind unwanted remnants, while electrochemical prelithiation lacks practical compatibility.^[^ ^15^, ^16^ ^]^ Among these methods, chemical prelithiation, which involves the use of a Li‐aromatic hydrocarbon (arene) complex (LAC) solution with strong reducing capability (that drives Li^+^ ions into the electrode material) along with an oxidation reaction of the arene, has many advantages.^[^ ^17^, ^18^ ^]^ This method is characterized by its simplicity, rapidity, high yield, high safety, and great prelithiation homogeneity (since it is a solution‐based process).^[^ ^6^, ^8^ ^]^ Chemical prelithiation is a spontaneous redox reaction that happens between the LAC and electrode. Therefore, no additional electricity, heat, or force needs to be applied and the whole process is easily scalable. Chemical prelithiation has shown great potential for industrial application with a roll‐to‐roll design.^[^ ^10^ ^]^ However, the LAC solution recipe, which includes various arene compounds and solvents, needs further optimization to achieve facile and cost‐effective prelithiation.

The electron affinity of various polycyclic aromatic hydrocarbons (PAHs) is distinct. Different PAHs thus show different prelithiation abilities.^[^ ^19^ ^]^ Qu et al. proposed an organolithium compound of 9,9‐dimetly‐9H‐fluorene, which showed a low redox potential of ≈0.18 V (vs Li^+^/Li).^[^ ^20^ ^]^ This allows fast and effective prelithiation of the electrode. The prelithiation capability can be tuned by introducing a functional group onto the aromatic ring. Lee et al. modified the molecular structure of biphenyl (BP) by introducing electron‐donating alkyl groups at various positions on the benzene ring.^[^ ^17^ ^]^ This increased the electron density and thus decreased the electron affinity and redox potential of the PAHs. Accordingly, one methyl substitution at the ortho position (2‐methyl BP) and four methyl groups at both the meta and para positions (3,3′,4,4′‐tetramethyl BP) led to an optimal prelithiation power for Si/SiO*_x_* electrodes. In another study, Qian et al. engineered naphthalene (Naph) with various functional groups at the α position.^[^ ^21^ ^]^ Among the PAHs investigated, 1‐cyano Naph not only formed a superior SEI that contained a dense N‐containing organic outer layer and an inorganic LiF‐rich inner layer but also increased the initial Coulombic efficiency (ICE) of the SiO electrode to above 100%.

In addition to PAHs, the solvent in the LAC solution plays a critical role in determining prelithiation activity. The electron‐donating ability of the solvent is important. Using a solvent with a stronger electron‐donating ability leads to a lower redox potential of the LAC. Ai et al. used a modified tetrahydrofuran (THF) solvent, 2‐methyl‐tetrahydrofuran (2‐Me‐THF), to increase the tendency of donating electrons.^[^ ^22^ ^]^ Accordingly, Li‐BP/2‐Me‐THF had a clearly lower redox potential than that of Li‐BP/THF. As a result, the former solution exhibited superior lithiation capability for a graphite anode. Lee et al. conducted prelithiation experiments using various Li‐BP solutions with different solvents, whose solvation power increased in the order of tetrahydropyran < 2‐Me‐THF < THF < ethylene glycol dimethyl ether.^[^ ^18^ ^]^ When the strong solvent dimethyl ether was utilized, the formation of solvent‐separated ion pairs was predominant, leading to poor desolvation of Li^+^ ions and thereby the co‐intercalation of solvent molecules into the graphite structure. In contrast, when a weak solvent such as tetrahydropyran was utilized, the desolvation of the solvent dominated the process and thus the contact ion pairs and ion aggregates were the main species. As a consequence, better and more stable prelithiation performance for graphite/SiO*_x_* electrodes was obtained. The influence of the LAC solvent on prelithiation performance is not well understood. The optimal solvent for various PAHs can be different. This paper thus examines the solvent effects for Li‐Naph‐based prelithiation solutions.

**Scheme 1:**
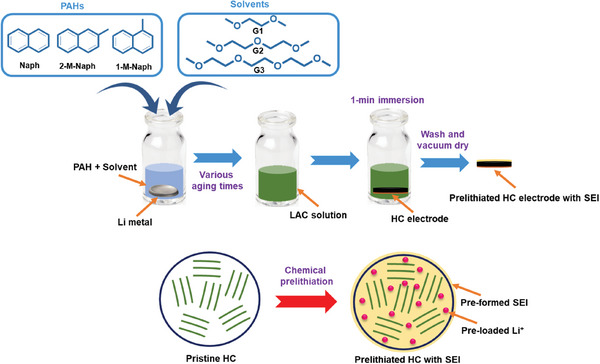
Schematic illustration of chemical prelithiation process developed in this study via optimization of PAH, solvent, and aging time.

In the present work, considering practical applications, inexpensive and readily available PAHs and solvents are used (i.e., complex and synthetic compounds are avoided). Herein, we employ HC as a model anode. HC has a large interplanar distance (0.36–0.38 nm), which facilitates Li^+^ transport and enables a higher specific capacity than that of graphite. Moreover, HC is more stable than Si‐based materials in terms of electrochemical reversibility. Although these advantages are desirable for LIBs, the low ICE of HC has long been a limiting factor. Therefore, HC is adopted to investigate three aspects of chemical prelithiation, as illustrated in **Scheme** **1**. In the first part of this study, various inexpensive PAHs, namely Naph, 2‐methylnaphthalene (2‐M‐Naph), and 1‐methylnaphthalene (1‐M‐Naph), are compared. The effects of methyl substitution and its position on the Naph ring are for the first time examined. In the second part, affordable and readily available solvents with low viscosity, namely dimethyl ether (G1), diethylene glycol dimethyl ether (G2), and triethylene glycol dimethyl ether (G3), are used to prepare LAC solutions. Their prelithiation performance is compared. The Li/PAH/solvent interaction time in the first two parts was 10 h. In the third part, the effects of the interaction time between Li metal, Naph, and solvent (e.g., the aging time of the LAC solution) on prelithiation power are investigated. Wu et al. found that 10 and 60 min of interaction time between Li metal and 4,4‐dimethylbiphenyl in THF solvent led to different prelithiation capabilities. The resulting electrodes showed distinct impedance and charge‐discharge properties.^[^ ^23^ ^]^ However, the interaction time for the Naph/ether system has not been studied. Thus, in this study, the effects of aging time (2, 4, 6, 8, 10, and 12 h) on the ICE, rate capability, and cycling stability of prelithiated HC electrodes are systematically analyzed. The prelithiation time (i.e., electrode immersion time in LAC solution) is fixed at 1 min for all LAC solution recipes, regardless of the PAH type, solvent type, and interaction time, in consideration of practical applications. The target of this work is to develop an effective chemical prelithiation protocol for boosting the performance of LIB anodes with low charge‐discharge Coulombic efficiency.

## 2 Results and Discussion

**Figure 1:**
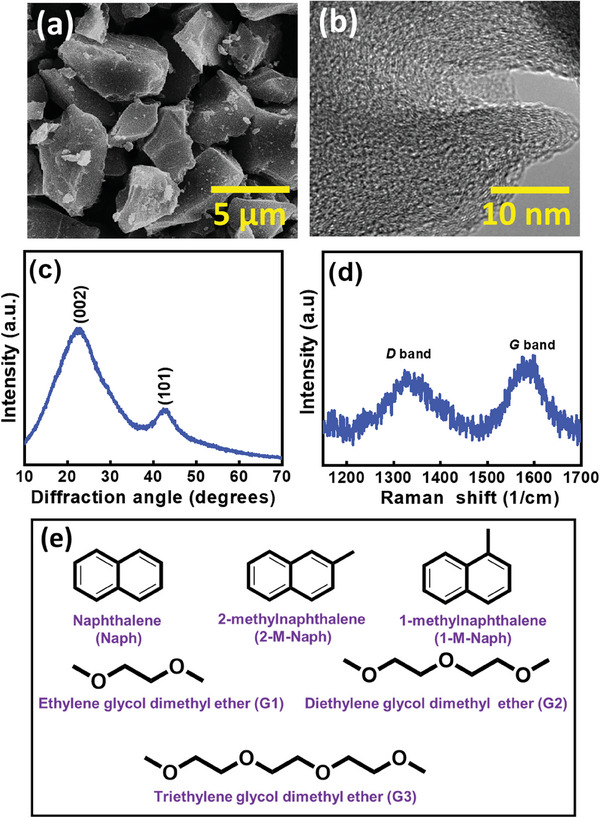
a) SEM image, b) TEM image, c) XRD pattern, and d) Raman spectrum of HC powder. e) Molecular structures of PAHs and solvents used in this study.

**Figure** **1a** shows the scanning electron microscopy (SEM) morphology of the HC used in this work. The HC has an irregular shape, with a particle size ranging from 5 to 10 µm. The high‐resolution transmission electron microscopy (TEM) image in Figure 1b shows the typical short‐range ordered structure of HC. The X‐ray diffraction (XRD) pattern in Figure 1c reveals the low crystallinity of HC. The broad peaks located at diffraction angles of ≈23° and ≈43° are associated with the (002) and (101) plane diffraction of non‐graphitic carbon, respectively. Based on these diffraction angles, the interplanar spacing *d* between the carbon layers was calculated to be ≈0.385 nm. The Brunauer–Emmett–Teller surface area of the HC was measured to be ≈6 m^2^ g^−1^. Raman spectroscopy was used to examine the carbon bonding characteristics. The obtained spectrum, shown in Figure 1d, exhibits a *D* band at ≈1330 cm^−1^ and a *G* band at ≈1580 cm^−1^. The former is associated with defective carbon bonding and the latter results from the Raman‐allowed in‐plane vibration of sp^2^ carbon bonds.^[^ ^24^ ^]^

### 2.1 Tailoring of Molecular Structures of Naphthalene for Prelithiation of HC Electrodes

**Figure 2:**
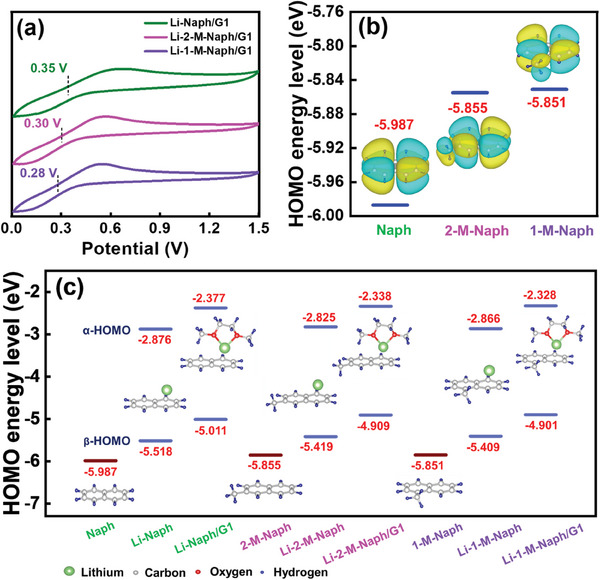
a) CV curves recorded in various LAC solutions at Pt electrodes. b) Electronic structures and HOMO energy levels of Naph, 2‐M‐Naph, and 1‐M‐Naph estimated using DFT calculations. c) HOMO levels of Naph compounds at various coordination states. The vacuum level is aligned to 0 eV.

To trigger chemical lithiation, it is crucial to adjust the redox potential of the reagent molecules to match the required value. Grafting various groups can change the electron density of the aromatic ring, resulting in a change in electron affinity and thus the redox potential.^[^ ^17^ ^]^ To clarify the effects of the tailored methyl group, 2‐M‐Naph and 1‐M‐Naph were compared to Naph (the molecular structures of these PAHs are shown in Figure 1e). As shown in **Figure** **2**a, all LAC solutions exhibit analogous cyclic voltammetry (CV) features at Pt electrodes, showing a pair of redox peaks, which indicates that the PAHs have similar electrochemical properties. Compared to Naph, the half‐wave potentials (*E*_1/2_) of its derivatives are negatively shifted; the degree of this potential shift depends on the methyl group position. The *E*_1/2_ values for Li‐Naph/G1, Li‐2‐M‐Naph/G1, and Li‐1‐M‐Naph/G1 solutions are 0.35, 0.30, and 0.28 V, respectively. Li‐1‐M‐Naph/G1 solution has the lowest potential and thus the highest prelithiation capability. Introducing an electron‐donating methyl group into the benzene ring can increase electron density and thus decrease electron affinity and redox potential.^[^ ^17^, ^21^ ^]^ The highest occupied molecular orbital (HOMO) energy levels of Naph, 2‐M‐Naph, and 1‐M‐Naph were estimated using density functional theory (DFT) calculations. The structural details and HOMO energy values are shown in Figure 2b, which indicates that the HOMO level of Naph (−5.987 eV) increases with the addition of methyl groups (i.e., −5.855 eV for 2‐M‐Naph and −5.851 eV for 1‐M‐Naph). As shown in Figure 2c, when the Naph compounds react with Li metal and coordinate with G1 molecules, the HOMO levels increase, reflecting an increase in reducing power. The formed Li‐Naph/G1, Li‐2‐M‐Naph/G1, and Li‐1‐M‐Naph/G1 complexes show different α/β HOMO energy levels (−2.377/−5.011 eV, −2.338/−4.909 eV, and −2.328/−4.901 eV, respectively). It is noted that regardless of the α and β levels and coordination states, the HOMO energy increases in the order of Naph < 2‐M‐Naph < 1‐M‐Naph, indicating that Li‐1‐M‐Naph/G1 solution has the highest tendency to release electrons for the prelithiation of an electrode. The DFT calculation results well coincide with the *E*_1/2_ data shown in Figure 2a.

**Figure 3:**
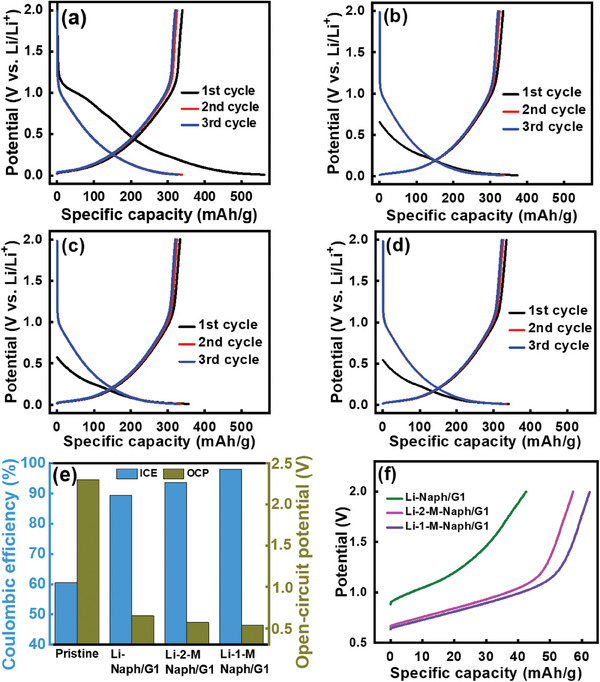
Initial charge‐discharge curves of a) pristine, b) Li‐Naph/G1, c) Li‐2‐M‐Naph/G1, and d) Li‐1‐M‐Naph/G1 HC electrodes measured at 50 mA g^−1^. e) ICE and OCP values of various HC electrodes. f) Direct‐discharge curves of various prelithiated HC electrodes measured at 50 mA g^−1^.

To evaluate the prelithiation performance, we immersed the HC electrodes in various LAC solutions. **Figure** **3**a‐d shows the initial three charge‐discharge profiles of the pristine and prelithiated HC electrodes measured at 50 mA g^−1^. The potential sloping region (i.e., 1.1–0.1 V) during charging (i.e., “lithiation” in this work) corresponds to Li^+^ insertion between carbon layers, whereas the low potential plateau (below 0.1 V) is associated with micropore filling with Li^+^.^[^ ^25^ ^]^ The ICE value of the pristine HC was 61% and those of the electrodes prelithiated with Li‐Naph/G1, Li‐2‐M‐Naph/G1, and Li‐1‐M‐Naph/G1 solutions were 89.4%, 93.5%, and 98.0%, respectively. Figure 3e summarizes the variations of ICE and open‐circuit potential (OCP) values of the HC electrodes. The prelithiated HC electrodes showed a clearly lower OCP than that of the pristine electrode. This reflects the SEI formation and insertion of Li^+^ into the HC that happened during the prelithiation process. The electrode treated with Li‐1‐M‐Naph/G1 shows the lowest OCP and highest ICE, which is consistent with the prediction based on the *E*_1/2_ measurement and DFT calculations (see Figure 2). The rise in the ICE is of great significance for practical LIBs. Because active Li^+^ loss is reduced, the cyclable Li^+^ and energy density of the cell are increased.^[^ ^22^, ^26^ ^]^

Figure S1a (Supporting Information) shows the XRD patterns of the pristine and Li‐1‐M‐Naph/G1‐prelithiated HC samples. Upon prelithiation, the diffraction intensity significantly decreases, implying Li^+^ insertion into the HC lattice.^[^ ^27^ ^]^ Figure S1b (Supporting Information) compares the Raman spectra of the two samples. As shown, the *D*‐to‐*G*‐band intensity ratio decreases after prelithiation. This is consistent with previously reported results for HC that uptakes Li^+^ and undergoes structural variation.^[^ ^28^, ^29^ ^]^ Furthermore, Figure S1c (Supporting Information) shows that a uniform and robust SEI formed on the prelithiated HC.

Figure S2a,b (Supporting Information) shows the initial three CV scans of the pristine and Li‐1‐M‐Naph/G1 prelithiated HC electrodes, respectively. For the pristine electrode, an irreversible reduction current appears in the first cathodic scan below 1 V, which is associated with electrolyte decomposition and SEI formation.^[^ ^30^, ^31^ ^]^ This reduction signal gradually decreases and the oxidation current slightly increases over subsequent cycles. In contrast, the initial three CV curves of the prelithiated HC electrode almost overlap, with the cathodic total charge and anodic total charge being nearly the same. No irreversible reaction was observed, indicating that a well‐developed and protective SEI layer formed on the electrode prior to the CV scan. In addition, the CV redox peaks of the prelithiated HC electrode appear to be narrower and sharper compared to those of the pristine HC. This implies that the SEI that formed during the prelithiation process is more Li^+^ conductive than the conventional SEI that formed during the CV scan. In the chemical prelithiation, besides SEI formation, the insertion of Li^+^ into the HC lattice took place. To evaluate the amount of Li^+^ that intercalated into the HC during the prelithiation, the electrodes were directly delithiated at 50 mA g^−1^ without first charging. The obtained data are shown in Figure 3f. The measured capacities of the HC electrodes prelithiated in Li‐Naph/G1, Li‐2‐M‐Naph/G1, and Li‐1‐M‐Naph/G1 were 42, 57, and 62 mAh g^−1^, respectively. These results confirm the superior prelithiation power of Li‐1‐M‐Naph/G1 solution. According to the electrode OCP values and the capacities shown in Figure 3f, the Li^+^ insertion reactions between carbon layers took place during the prelithiation process, whereas micropore filling did not happen.

**Figure 4:**
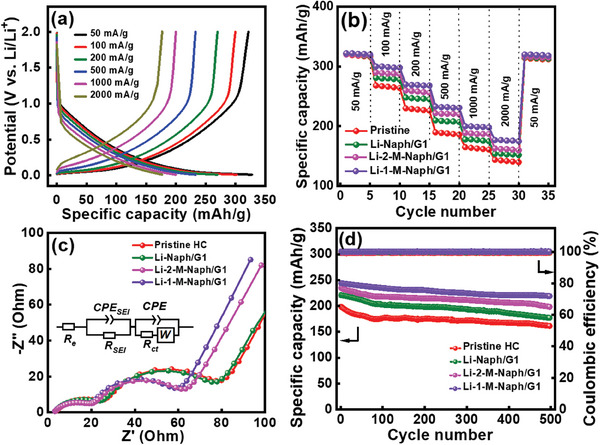
a) Charge–discharge curves of Li‐1‐M‐Naph/G1 HC electrode measured at various current rates. b) Comparative rate performance of various HC electrodes. c) EIS data of various HC electrodes measured after conditioning cycles (inset shows equivalent circuit). d) Cycling stability data of various HC electrodes measured at 400 mA g^−1^ for 500 cycles.

The rate capabilities of various HC electrodes were evaluated through galvanostatic charging/discharging at various current rates (from 50 to 2000 mA g^−1^). **Figure** **4**a shows the data for the HC electrode prelithiated in Li‐1‐M‐Naph/G1 solution. The charge‐discharge curves of the pristine HC and other prelithiated HC electrodes are shown in Figure S3 (Supporting Information). As compared in Figure 4b, at a low rate of 50 mA g^−1^, all electrodes exhibit similar specific capacities of ≈320 mAh g^−1^. However, the prelithiated HC electrodes have enhanced rate capability, as shown in Table S1 (Supporting Information). The reversible capacities of the pristine, Li‐Naph/G1, Li‐2‐M‐Naph/G1, and Li‐1‐M‐Naph/G1 HC electrodes were 144, 154, 163, and 177 mAh g^−1^, respectively, at 2000 mA g^−1^. These results suggest that chemical prelithiation produces a relatively conductive SEI, thereby facilitating Li^+^ transport across the interface and increasing the electrode high‐rate performance. Electrochemical impedance spectroscopy (EIS) was conducted for the HC electrodes after two conditioning cycles to analyze the impedance characteristics; the obtained data are shown in Figure 4c. The spectra consist of a semicircle (related to SEI) at high frequency, another semicircle (related to charge transfer at the interface) at medium frequency, and a sloping line (associated with Li^+^ diffusion in the electrode) at low frequency.^[^ ^32^ ^]^ The impedance characteristics can be described using the equivalent circuit shown in the figure inset, where *R*_e_, *R*_SEI_, *R*_ct_, *CPE*_SEI_, *CPE*_int_, and *W* represent the electrolyte resistance, SEI resistance, charge transfer resistance, SEI constant‐phase element, interfacial constant‐phase element, and Warburg impedance, respectively.^[^ ^33^ ^]^ The *R*_SEI_ and *R*_ct_ values of various HC electrodes are shown in **Table** **1**. The SEI on the Li‐1‐M‐Naph/G1 electrode is the most conductive and most facilitates the charge transfer reaction, leading to the best rate capability.

**Table 1:** Summary of electrochemical properties of pristine HC and prelithiated HC electrodes obtained using various LAC solutions.

	Pristine HC	Li‐Naph/G1	Li‐2‐M‐Naph/G1	Li‐1‐M‐Naph/G1	Li‐1‐M‐Naph/G2	Li‐1‐M‐Naph/G3
OCP [V]	2.3	0.65	0.57	0.54	0.88	0.93
ICE [%]	60.5	89.4	93.5	98.0	79.3	75.9
Direct delithiation capacity [mAh g^−1^]	0	42	57	62	28	17
Capacity at 50 mA g^−1^ [C_50_, mAh g^−1^]	320	321	320	322	320	321
Capacity at 2000 mA g^−1^ [C_2000_, mAh g^−1^]	144	154	163	177	125	109
High rate retention [C_50_/C_2000_] [%]	45	48	51	55	39	34
Capacity retained after 500 cycles [%]	80	81	84	90	80	78
*R*_SEI_ [Ω]	25	23	21	18	33	39
*R*_ct_ [Ω]	57	52	45	40	80	87

Figure 4d shows the cycling stability data of the HC electrodes measured at 400 mA g^−1^ for 500 cycles. The electrodes prelithiated with Li‐Naph/G1, Li‐2‐M‐Naph/G1, and Li‐1‐M‐Naph/G1 solutions show capacity retention ratios of 81%, 84%, and 90%, respectively, compared to a capacity retention ratio of 80% for the pristine HC electrode after the same number of cycles. This can be attributed to the more robust and protective SEI layer that formed in the Li‐1‐M‐Naph/G1 solution. Figure S4 (Supporting Information) shows SEM images of the pristine and Li‐1‐M‐Naph/G1 HC electrodes after cycling. Numerous deposits, associated with dead Li and accumulated SEI, were observed on the pristine HC. In contrast, the surface of the Li‐1‐M‐Naph/G1 HC electrode was relatively smooth, reflecting ideal coverage by a thin and dense SEI layer. It is speculated that the continuous irreversible reactions on the pristine HC electrode contributed to the more pronounced capacity deterioration. Figure S5 (Supporting Information) further verifies the long‐term stability of the Li‐1‐M‐Naph/G1 HC electrode, which demonstrates 87% capacity retention after 500 cycles at an elevated temperature of 50 °C.

**Figure 5:**
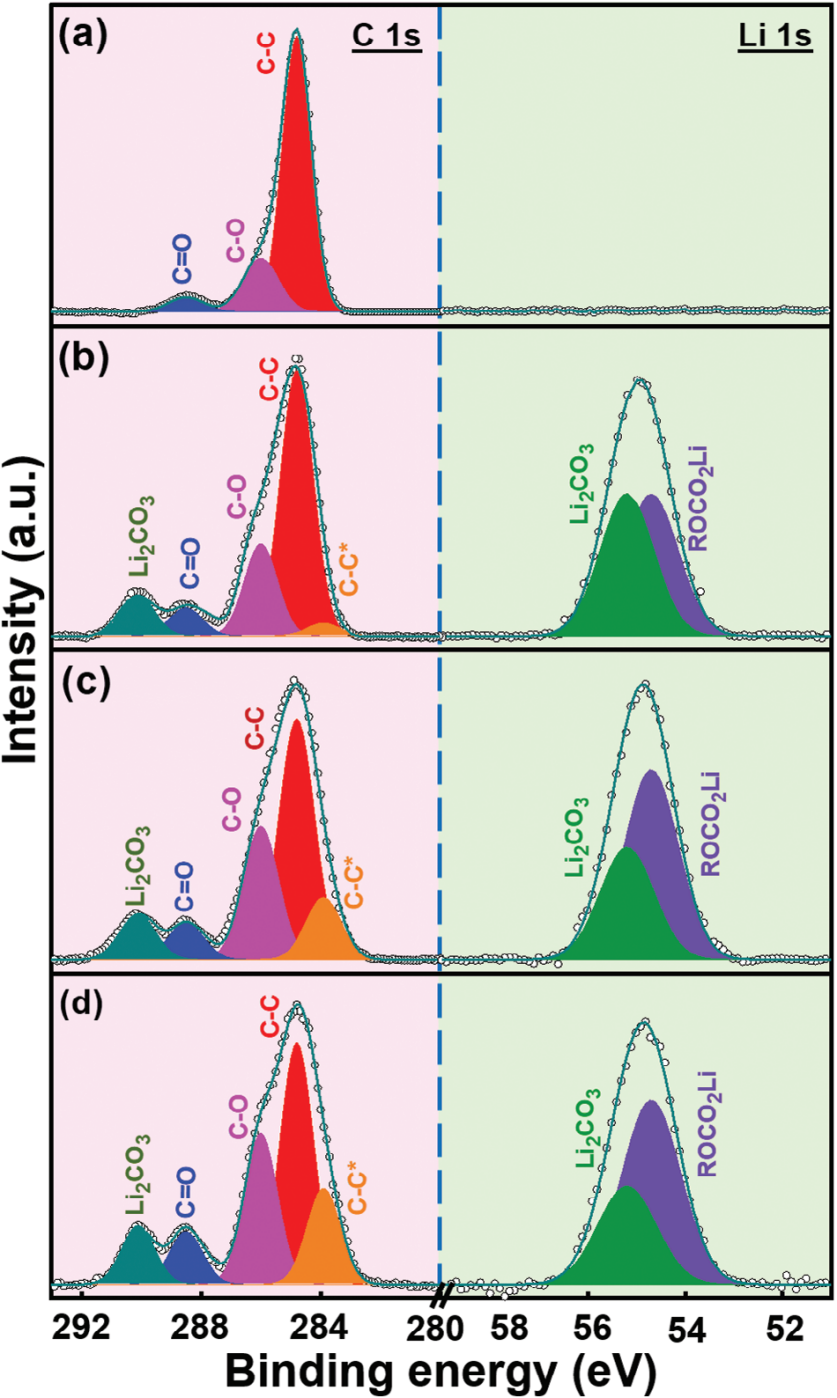
XPS C 1s and Li 1s spectra of a) pristine, b) Li‐Naph/G1, c) Li‐2‐M‐Naph/G1, and d) Li‐1‐M‐Naph/G1 HC electrodes.

X‐ray photoelectron spectroscopy (XPS) analyses were performed to gain further insight into the chemistry of the SEI on the HC electrodes. **Figure** **5**a shows the C 1s and Li 1s spectra of the pristine electrode. Besides the C−C sp^2^ peak at 284.8 eV, the signals at 286.0 and 288.4 eV are associated with C─O and C═O bonding, respectively, which are attributed to the existence of the sodium polyacrylate binder and surface functional groups on the electrode.^[^ ^21^, ^34^ ^]^ After prelithiation in Li‐Naph/G1, Li‐2‐M‐Naph/G1, and Li‐1‐M‐Naph/G1 solutions, Li_2_CO_3_ signals clearly appear in the XPS spectra (Figure 5b‐d), confirming the formation of surface layers on the electrodes. It is found that the C─O and C═O peak intensities increase after prelithiation, which is related to the newly created organic ROCO_2_Li species in the SEI. Furthermore, an additional C─C* peak at 283.9 eV appears after prelithiation, which indicates an electron‐rich state.^[^ ^35^ ^]^ During the prelithiation process, electrons are transferred from the aromatic anion radicals to the HC,^[^ ^23^ ^]^ forming this electron‐rich configuration. The C─C* peak intensity increases in the order of Li‐Naph/G1 < Li‐2‐M‐Naph/G1 < Li‐1‐M‐Naph/G1, which is in line with the electron‐donating capability of the PAHs revealed in the CV and DFT data (Figure 2). Figure 5 also reveals that the ratio of organic ROCO_2_Li to inorganic Li_2_CO_3_ increases with increasing prelithiation power of the LAC solution (Li‐Naph/G1 < Li‐2‐M‐Naph/G1 < Li‐1‐M‐Naph/G1). Although the inorganic component can provide great passivation ability and high mechanical strength,^[^ ^36^, ^37^ ^]^ it is brittle and can thus easily break during long‐term cycling (due to electrode volume variation) or high‐current‐rate operation. The organic content in the SEI can serve as a glue to withstand the strain during cycling.^[^ ^21^, ^38^, ^39^ ^]^ The balanced organic and inorganic composition of the SEI formed on the Li‐1‐M‐Naph/G1 HC electrode led to its superior rate capability and cyclability.

### 2.2 Solvent Selection for Prelithiation of HC Electrodes

**Figure 6:**
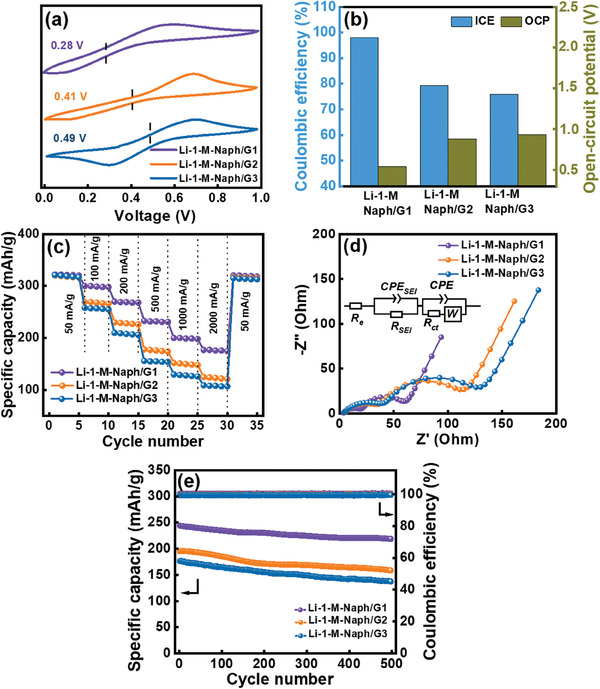
a) CV curves recorded in various LAC solutions at Pt electrodes. b) ICE and OCP values of various prelithiated HC electrodes. c) Comparative rate performance of various prelithiated HC electrodes. d) EIS data of various prelithiated HC cells measured after conditioning cycles (inset shows equivalent circuit). e) Cycling stability data of various prelithiated HC electrodes measured at 400 mA g^−1^ for 500 cycles.

Modulating the solvent of the LAC solution can optimize prelithiation performance.^[^ ^18^, ^22^ ^]^ It has been reported that a solvent with weak Li^+^ solvation power and strong electron‐donating ability to aromatic anion radicals is preferred.^[^ ^18^ ^]^ In this section, we adopt Li‐1‐M‐Naph/G2 and Li‐1‐M‐Naph/G3 to prelithiate HC electrodes (the molecular structures of the solvents are illustrated in Figure 1e). As shown in the CV curves in **Figure** **6**a, the *E*_1/2_ values for Li‐1‐M‐Naph/G2 and Li‐1‐M‐Naph/G3 are 0.41 and 0.49 V, respectively, which are higher than that (0.28 V) for Li‐1‐M‐Naph/G1 solution. The longer chain length of G2 and G3 (vs G1) may cause steric hindrance for (1‐M‐Naph)^•−^ radicals to transfer electrons to the electrodes, leading to the higher *E*_1/2_ values.

Figure S6 (Supporting Information) shows the initial three charge‐discharge profiles of the HC electrodes prelithiated in Li‐1‐M‐Naph/G2 and Li‐1‐M‐Naph/G3 solutions. As shown in Figure 6b, the ICE values are 79.3% and 75.9%, respectively, and the OCP values are 0.88 and 0.93 V, respectively. All properties are worse than those obtained for Li‐1‐M‐Naph/G1 solution. Moreover, as shown in Figure S7 (Supporting Information), the direct discharge tests indicate that the intercalated Li^+^ amounts in the HC electrodes prelithiated in Li‐1‐M‐Naph/G2 and Li‐1‐M‐Naph/G3 solutions are 28 and 17 mAh g^−1^, respectively (vs 62 mAh g^−1^ for the Li‐1‐M‐Naph/G1 HC). Our data confirm that the prelithiation ability of the LAC solution indeed depends on the solvent. As mentioned above, the steric hindrance effects can alter the *E*_1/2_ values. In addition, the G2 and G3 molecules have three and four oxygen atoms, respectively, to coordinate with Li^+^ (the G1 molecule has two oxygen atoms).^[^ ^40^, ^41^ ^]^ This makes it more difficult for the Li^+^ ions to be released and inserted into the HC electrodes, decreasing the prelithiation power. Moreover, the longer chain length of G2 and G3 increases the solution viscosity and thus hinders Li^+^ mobility toward the electrodes. The experimental results indicate that for a similar molecular structure and chemistry, the solvent with a smaller molecular weight will have better prelithiation capability.

Figure S8a,b (Supporting Information) shows the charge‐discharge curves of the HC electrodes prelithiated in Li‐1‐M‐Naph/G2 and Li‐1‐M‐Naph/G3 solutions, respectively, measured at various specific currents. Figure 6c compares the rate capability of various prelithiated HC electrodes. At 50 mA g^−1^, the specific capacities of the three electrodes are similar. However, when the rate is increased to 2000 mA g^−1^, the measured capacities of the Li‐1‐M‐Naph/G2 and Li‐1‐M‐Naph/G3 HC electrodes are 125 and 109 mAh g^−1^, respectively, compared to 177 mAh g^−1^ for the Li‐1‐M‐Naph/G1 HC electrode. As shown in Table S2 (Supporting Information), the capacity retentions of the G1‐, G2‐, and G3‐prelithiated electrodes at 2000 mA g^−1^ (compared to those found at 50 mA g^−1^) are 55%, 39%, and 34%, respectively. The EIS data in Figure 6d show that the *R*_SEI_ and *R*_ct_ values for the Li‐1‐M‐Naph/G2 and Li‐1‐M‐Naph/G3 electrodes are clearly higher than those for the Li‐1‐M‐Naph/G1 electrode (see Table 1). The Li‐1‐M‐Naph/G3 HC electrode having the highest *R*_SEI_ and *R*_ct_ values explains its inferior rate capability. Figure 6e shows that the capacity retention ratios after 500 cycles for the Li‐1‐M‐Naph/G2 and Li‐1‐M‐Naph/G3 HC electrodes are 80% and 78%, respectively, both lower than that (90%) for the Li‐1‐M‐Naph/G1 electrode. Presumably, due to the lower prelithiation power of the G2 and G3 solutions, a robust and protective SEI may not have sufficiently developed, leading to lower cyclability. This argument is supported by the postmortem SEM images in Figure S9 (Supporting Information), which show more side reaction products accumulated on the G2 and G3 HC electrodes.

### 2.3 Effects of LAC Aging Time on Prelithiation Performance

**Figure 7:**
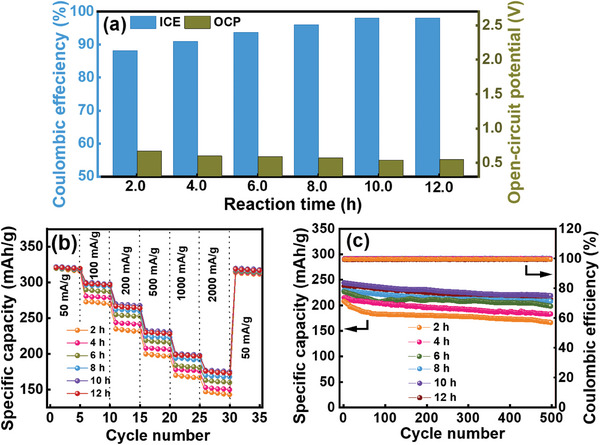
a) ICE and OCP values, b) comparative rate performance, and c) cycling stability data (measured at 400 mA g^−1^) of various HC electrodes prelithiated using Li‐1‐M‐Naph/G1 solution with different aging times.

Chemical prelithiation involves using active lithiation reagents based on the reaction of Li metal and PAHs.^[^ ^6^, ^20^ ^]^ An electron‐transfer reaction occurs when Li metal is added to a solvent (such as G1) with a PAH compound (such as 1‐M‐Naph). For Li‐1‐M‐Naph/G1, one Li atom loses an electron to become Li^+^, which is solvated by G1 to form Li(G1)*_n_* ^+^, while an 1‐M‐Naph molecule accepts this electron to form an anion radical (1‐M‐Naph)^•−^, in which the electron is delocalized in the aromatic ring.^[^ ^23^ ^]^ The electron will be transferred to the electrode to trigger Li^+^ (in the LAC solution) reduction and thus the prelithiation process (please see the discussion based on DFT calculations in Figure S10, Supporting Information). The effects of the mixing time between Li metal and 1‐M‐Naph in G1 solvent (e.g., the aging time of the LAC solution) on the prelithiation performance are unknown and thus investigated here. Aging times of 2, 4, 6, 8, 10, and 12 h were adopted before the HC prelithiation process (1 min of immersion). Figure S11 (Supporting Information) shows the initial charge‐discharge curves (measured at 50 mA g^−1^) of the HC electrodes prelithiated using Li‐1‐M‐Naph/G1 solution with various aging times. As shown in **Figure** **7**a, the OCP values of the HC electrodes decrease with increasing aging time and become saturated after 10 h. The ICE values of the HC electrodes prelithiated with LAC solution with an aging time of 2, 4, 6, 8, 10, and 12 h are 88.2%, 91.0%, 93.7%, 96.1%, 98.0%, and 98.1%, respectively. During aging, the Li^+^ cations and (1‐M‐Naph)^•−^ anions in the LAC solution could sequentially form G1‐separated ion pairs, contact ion pairs, and ion aggregates. Figure S12 (Supporting Information) shows the ^7^Li nuclear magnetic resonance (NMR) data of the LAC solution with various aging times. The noticeable upfield shift of the resonance peak confirms the formation of contact ion pairs and ion aggregates over time.^[^ ^42^, ^43^ ^]^ The prelithiation capability of the LAC solution thus varied with aging time. After 10 h, the peak position remained mostly unchanged, reflecting that the solution coordination structure became stable.

**Figure 8:**
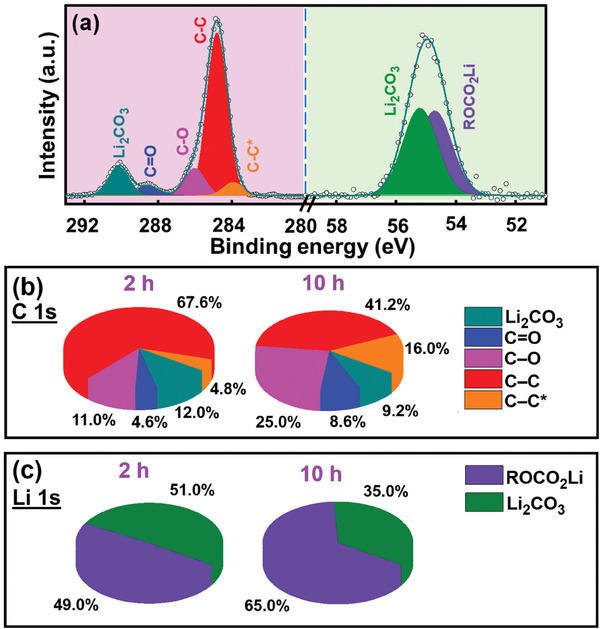
a) XPS C 1s and Li 1s spectra of HC electrode prelithiated using Li‐1‐M‐Naph/G1 solution with 2‐h aging. Difference in constituent species obtained in b) C 1s and c) Li 1s spectra for electrodes prelithiated using Li‐1‐M‐Naph/G1 solution with 2‐h and 10‐h aging.

The rate capability of various prelithiated HC electrodes was evaluated. The obtained data are shown in Figure S13 (Supporting Information). As compared in Figure 7b and Table S3 (Supporting Information), the electrode high‐rate performance increases with increasing aging time up to 10 h and becomes stable afterwards. Figure 7c shows that the capacity retention ratios after 500 charge–discharge cycles of the HC electrodes prelithiated with LAC solution with an aging time of 2, 4, 6, 8, 10, and 12 h are 81%, 85%, 88%, 89%, 90%, and 89%, respectively. As before, 10‐h aging is needed to reach the optimal performance. To examine the reasons for the electrode performance difference, an XPS study was performed on the HC electrode prelithiated using LAC solution with 2‐h aging. The obtained data in **Figure** **8**a were compared to the data in Figure 5d (HC electrode prelithiated using LAC solution with 10‐h aging) to examine the effects of aging time. The 2‐h HC electrode shows a much lower intensity of the electron‐rich C─C* peak. This suggests that the formation of (1‐M‐Naph)^•−^ anion radicals is still insufficient after 2 h, leading to less electron transfer to the HC electrode. Moreover, as shown in Figure 8b,c, the C─O and C═O (in C 1s spectrum) and ROCO_2_Li (in Li 1s spectrum) species on the 2‐h electrode are clearly less abundant than those on the 10‐h electrode. As shown in Figure S12 (Supporting Information), the ion pairs and aggregates preferentially form after 10‐h aging; therefore, more free G1 molecules exist in the LAC solution. The free G1 molecules more easily decompose compared to the solvated G1 molecules.^[^ ^44^, ^45^ ^]^ As a consequence, the 10‐h electrode surface is relatively rich in organic species. Since the organic components can effectively accommodate the volume change and maintain SEI integrity,^[^ ^21^, ^38^, ^39^ ^]^ the 10‐h HC electrode showed superior charge–discharge properties (see Figure 7). We believe that besides the PAH compound and LAC solvent selection, the LAC aging time is a crucial factor that affects the SEI chemistry and thus the electrochemical performance and cycle life of prelithiated electrodes.

Table S4 (Supporting Information) compares the prelithiation performance of the proposed LAC solution with data reported in the literature for HC electrodes. The LAC solution developed in this work is highly effective for fast prelithiation and significantly improves the electrode rate capability and cycling stability. In addition, the PAH and solvent used in our LAC solution are readily available and inexpensive (as shown in Table S5, Supporting Information). We also used the Li‐1‐M‐Naph/G1 solution to prelithiate an SiO*_x_* anode. The data in Figure S14 (Supporting Information) indicate that the LAC solution can clearly increase the ICE and high rate performance of the SiO*_x_* electrode. Figure S15 (Supporting Information) shows the charge–discharge curves of Li‐ion full cells consisting of LiNi_0.6_Mn_0.2_Co_0.2_O_2_ cathodes and HC anodes without and with prelithiation (the anode‐to‐cathode capacity ratio is 1.15:1). Using the HC without prelithiation resulted in relatively poor performance because the HC consumed lots of cyclable Li^+^ ions in the first cycle. In contrast, with the prelithiated HC, the full cell exhibited superior charge‐discharge properties. The efficacy of the Li‐1‐M‐Naph/G1 prelithiation solution is thus validated.

## 3 Conclusion 

A cost‐effective chemical prelithiation solution was developed for an LIB anode. It was found that introducing an electron‐donating methyl group at the alpha position on the Naph ring substantially decreased the *E*_1/2_ value from 0.35 to 0.28 V (vs Li^+^/Li). DFT calculations confirmed that the HOMO energy level of Naph (regardless of the coordination status) increases with the addition of the methyl group, reflecting an increase in reducing power. For solvent selection, G2 and G3 have more oxygen atoms (compared to G1) to coordinate with Li^+^ ions. This makes it more difficult for the Li^+^ ions to be released and inserted into the HC electrode. Moreover, due to the lower viscosity and less steric hindrance of G1, it clearly outperformed G2 and G3 as a LAC solvent in terms of prelithiation capability. The Li‐1‐M‐Naph/G1 solution not only prelithiated the HC to the highest degree but also created an SEI with a balanced organic and inorganic composition. This unique SEI minimized the *R*_SEI_ and *R*_ct_ values, leading to the superior rate capability of the prelithiated HC electrode compared to that of a pristine HC electrode. This pre‐formed SEI also reduced the dead Li and accumulated SEI, increasing the cyclability of the electrode. It was found that the aging time greatly affected the prelithiation capability of the LAC solution; it took ≈10 h to reach saturation for Li‐1‐M‐Naph/G1. According to XPS data, insufficient aging caused less electron transfer and an inorganic‐species‐rich SEI, which was relatively brittle and susceptible to electrode volume‐variation‐induced damage. We believe that our findings will provide guidelines for better LAC recipe design. The proposed optimized chemical prelithiation protocol can be adopted to cost‐effectively increase the performance of LIBs.

## 4 Experimental section

### Materials

Naph (Across Organics, 99%), 2‐M‐Naph (Across Organics, 99%), and 1‐M‐Naph (Alfa Aesar, 99%) were used as received. G1 (Across Organics, 99%), G2 (Across Organics, 99%), and G3 (Across Organics, 99%) were dried over fresh molecular sieves for two days to remove residual water prior to use. All chemicals were stored in an argon‐filled glovebox (Vigor Tech. Co. Ltd.) to prevent exposure to air. HC (Carbotron P, Kureha Co.) was used as received.

### Preparation of HC Electrodes and Prelithiation Solution

The electrode slurry was composed of 80 wt.% HC, 10 wt.% Super P, and 10 wt.% sodium polyacrylate binder in deionized water. The slurry was cast onto Cu foil with a doctor blade and vacuum‐dried at 90 °C for 8 h. The resulting electrode was roll‐pressed and punched to match the required dimensions of a CR2032 coin cell. The HC mass loading was ≈5 mg cm^−2^. Prelithiation solutions were prepared by reacting Li metal with various PAHs in various ether solvents. In the first part of this study, Naph, 2‐M‐Naph, or 1‐M‐Naph along with Li metal were dissolved in G1 solvent to form three types of 0.5 M LAC solution, namely Li‐Naph/G1, Li‐2‐M‐Naph/G1, and Li‐1‐M‐Naph/G1. In the second part, Li metal and 1‐M‐Naph were dissolved in G2 and G3, respectively, to produce Li‐1‐M‐Naph/G2 and Li‐1‐M‐Naph/G3 (to compare with Li‐1‐M‐Naph/G1 from the first part). The effects of the solution solvent were investigated. The Li/PAH/solvent interaction time in the first two parts was 10 h. In the third part, the interaction time was varied from 2 to 12 h to optimize prelithiation performance. The prelithiation time (i.e., electrode immersion time in LAC solution) was fixed at 1 min for all LAC solution recipes. The prelithiated electrodes were washed and vacuum‐dried before further analyses. This prelithiation process was safe and can be conducted at ambient temperatures. Some precautions and operation measures can be found in the literature.^[^ ^13^, ^46^ ^]^

### Material Characterization

The sample crystallinity was examined using an X‐ray diffractometer (Bruker D8 Discover) with Cu K_α_ radiation (λ = 0.15418 nm) as the X‐ray source. The Raman spectrum was collected using a spectrometer (LabRAM HR 800) with an excitation laser wavelength of 633 nm. The morphologies and microstructures of the samples were characterized using SEM (JEOL 6701F) and TEM (JEOL F200). The surface chemistry of the electrodes was analyzed using XPS (Thermo Fisher Scientific ESCALAB 250Xi). Monochromatic Al K_α_ radiation (1486.6 eV) was adopted as the X‐ray source. After the prelithiation process, the electrodes were moved to the XPS chamber in an air‐tight vessel to avoid exposure to air. For ^7^Li NMR analysis, the LAC solution was sealed in an NMR tube in an argon‐filled glovebox and then transferred to the instrument. All the ^7^Li NMR shifts were referenced to 0.5 m LiCl in D_2_O as an external standard.

### Cell Assembly and Electrochemical Measurements

Li foil and a polyethylene membrane were used as the counter electrode and separator, respectively. An electrolyte composed of 1 m LiPF_6_ salt and ethylene carbonate/diethyl carbonate mixed solvent (1:1 by volume) was used. The CR2032 coin cells were assembled in an Ar‐filled glove box, where both the oxygen and moisture content levels were maintained at ≈0.1 ppm. CV and EIS measurements were carried out using a potentiostat (Biologic VSP‐300). The cell charge‐discharge properties (i.e., capacity, rate capability, and cycling stability) were evaluated using a battery tester (NEWARE CT‐4000) at 25 °C. For each condition, at least five parallel cells were measured. The performance deviation was typically within ≈3%. The reported data were the medians.

### Computational Methods

First‐principles calculations based on spin‐polarized DFT with the projector augmented wave method^[^ ^47^ ^]^ were conducted using the Vienna Ab initio Simulation Package (VASP).^[^ ^48^, ^49^ ^]^ The Perdew–Burke–Ernzerhof^[^ ^50^ ^]^ functional within the generalized‐gradient approximation^[^ ^51^ ^]^ was used to describe the exchange‐correlation interaction for structure optimization. The energy level calculations were performed with the screened hybrid functional HSE06 of Heyd, Ernzerhof, and Scuseria.^[^ ^52^, ^53^ ^]^ The cut‐off energy was set to 600 eV and the Brillouin zone was sampled with an 1 × 1 × 1 *k*‐point grid. The energy convergence criterion was set to 1.0 × 10^−5^ for all calculations. The atoms were allowed to relax until the force components were smaller than 0.01 eV Å^−1^.

### Statistical Analysis

The charge–discharge and EIS measurements of various electrodes were repeated at least five times to ensure validity. The data deviation was typically within 3% and the reported values were the medians. All XPS spectra were calibrated with the binding energy of the C 1s peak at 284.6 eV. The data fitting was done using XPSPEAK 4.1 software. Origin software (OriginLab) was used for data analysis and processing.

## Conflict of Interest

The authors declare no conflict of interest.

## Supporting information

Supporting Information

## Data Availability

The data that support the findings of this study are available from the corresponding author upon reasonable request.

## References

[1] M. Li , J. Lu , Z. Chen , K. Amine , Adv. Mater. 2018, 30, 1800561. 10.1002/adma.201800561 29904941

[2] F. Wu , J. Maier , Y. Yu , Chem. Soc. Rev. 2020, 49, 1569. 32055806 10.1039/c7cs00863e

[3] G. G. Eshetu , H. Zhang , X. Judez , H. Adenusi , M. Armand , S. Passerini , E. Figgemeier , Nat. Commun 2021, 12, 5459. 34526508 10.1038/s41467-021-25334-8 PMC8443554

[4] J. Patra , T. X. Nguyen , C. C. Tsai , O. Clemens , J. Li , P. Pal , W. K. Chan , C. H. Lee , H. Y. T. Chen , J. M. Ting , J. K. Chang , Adv. Funct. Mater. 2022, 32, 2110992.

[5] M. Okubo , S. Ko , D. Dwibedi , A. Yamada , J. Mater. Chem. A 2021, 9, 7407.

[6] Z. Huang , Z. Deng , Y. Zhong , M. Xu , S. Li , X. Liu , Y. Zhou , K. Huang , Y. Shen , Y. Huang , Carbon Energy 2022, 4, 1107.

[7] L. Sun , Y. Liu , J. Wu , R. Shao , R. Jiang , Z. Tie , Z. Jin , Small 2022, 18, 2102894. 10.1002/smll.202102894 34611990

[8] C. Xin , J. Gao , R. Luo , W. Zhou , Chem. ‐ Eur. J. 2022, 28, e202104282. 35137468 10.1002/chem.202104282

[9] R. Zhan , X. Wang , Z. Chen , Z. W. She , L. Wang , Y. Sun , Adv. Energy Mater. 2021, 11, 2101565.

[10] T. Jia , G. Zhong , Y. Lv , N. Li , Y. Liu , X. Yu , J. Zou , Z. Chen , L. Peng , F. Kang , Y. Cao , Green Energy Environ. 2023, 8, 1325.

[11] F. Wang , B. Wang , J. Li , B. Wang , Y. Zhou , D. Wang , H. Liu , S. Dou , ACS Nano 2021, 15, 2197. 33570903 10.1021/acsnano.0c10664

[12] Y. S. Su , J. K. Chang , Batteries 2022, 8, 99.

[13] F. Li , Y. Cao , W. Wu , G. Wang , D. Qu , Small Methods 2022, 6, 2200411. 10.1002/smtd.202200411 35680608

[14] X. Min , G. Xu , B. Xie , P. Guan , M. Sun , G. Cui , Energy Stor. Mater. 2022, 47, 297.

[15] W. Zhong , Z. Zeng , S. Cheng , J. Xie , Adv. Funct. Mater. 2024, 34, 2307860. 10.1002/adfm.202400023 PMC11469589 39399303

[16] H. Zhang , J. Cheng , H. Liu , D. Li , Z. Zeng , Y. Li , F. Ji , Y. Guo , Y. Wei , S. Zhang , T. Bai , X. Xu , R. Peng , J. Lu , L. Ci , Adv. Energy Mater. 2023, 13, 2300466.

[17] J. Jang , I. Kang , J. Choi , H. Jeong , K. W. Yi , J. Hong , M. Lee , Angew. Chem., Int. Ed. 2020, 59, 14473. 10.1002/anie.202002411 32400120

[18] J. Choi , H. Jeong , J. Jang , A. R. Jeon , I. Kang , M. Kwon , J. Hong , M. Lee , J. Am. Chem. Soc. 2021, 143, 9169. 34111352 10.1021/jacs.1c03648

[19] N. L. Holy , Chem. Rev. 1974, 74, 243.

[20] X. Zhang , H. Qu , W. Ji , D. Zheng , T. Ding , D. Qiu , D. Qu , J. Power Sources 2020, 478, 229067.

[21] Y. Li , Y. Qian , Y. Zhao , N. Lin , Y. Qian , Sci. Bull. 2022, 67, 636. 10.1016/j.scib.2021.12.010 36546125

[22] Y. Shen , X. Shen , M. Yang , J. Qian , Y. Cao , H. Yang , Y. Luo , X. Ai , Adv. Funct. Mater. 2021, 31, 2101181.

[23] H. Yue , S. Zhang , T. Feng , C. Chen , H. Zhou , Z. Xu , M. Wu , ACS Appl. Mater. Interfaces 2021, 13, 53996. 34732046 10.1021/acsami.1c16842

[24] J. Patra , B. R. Pan , M. H. Lin , C. Y. Su , S. W. Lee , T. Y. Wu , R. S. Dhaka , C. T. Hsieh , J. K. Chang , Electrochim. Acta 2022, 425, 140713.

[25] D. A. Stevens , J. R. Dahn , J. Electrochem. Soc. 2001, 148, A803.

[26] Y. Luo , Y. Deng , Y. Shen , H. Li , Y. Cao , X. Ai , Energy Technol. 2022, 10, 2200269.

[27] S. Alvin , H. S. Cahyadi , J. Hwang , W. Chang , S. K. Kwak , J. Kim , Adv. Energy Mater. 2020, 10, 2000283.

[28] H. Euchner , B. P. Vinayan , M. A. Reddy , M. Fichtner , A. Grob , J. Mater. Chem. A 2020, 8, 14205.

[29] S. Huang , Z. Li , B. Wang , J. Zhang , Z. Peng , R. Qi , J. Wang , Y. Zhao , Adv. Funct. Mater. 2018, 10, 1706294.

[30] X. Zhang , H. Qu , W. Ji , D. Zheng , T. Ding , C. Abegglen , D. Qiu , D. Qu , ACS Appl. Mater. Interfaces 2020, 12, 11589. 32056422 10.1021/acsami.9b21417

[31] R. Muruganantham , F. M. Wang , R. A. Yuwono , M. Sabugaa , W. R. Liu , Energy Fuels 2021, 35, 10878.

[32] P. Lurili , C. Brivio , V. Wood , J. Power Sources 2021, 505, 229860.

[33] W. Choi , H. C. Shin , J. M. Kim , J. Y. Choi , W. S. Yoon , J. Electrochem. Sci. Technol. 2020, 11, 1.

[34] Y. Shen , J. Qian , H. Yang , F. Zhong , X. Ai , Small 2020, 16, 1907602. 10.1002/smll.201907602 31990451

[35] A. Fujimoto , Y. Yamada , M. Koinuma , S. Sato , Anal. Chem. 2016, 88, 6110. 27264720 10.1021/acs.analchem.6b01327

[36] Q. Zhang , J. Pan , P. Lu , Z. Liu , M. W. Verbrugge , B. W. Sheldon , Y. T. Cheng , Y. Qi , X. Xiao , Nano Lett. 2016, 16, 2011. 26889564 10.1021/acs.nanolett.5b05283

[37] Y. Liu , D. Lin , P. Y. Yuen , K. Liu , J. Xie , R. H. Dauskardt , Y. Cui , Adv. Mater. 2017, 29, 1605531. 10.1002/adma.201605531 28032934

[38] S. Chen , Z. Wang , L. Wang , Z. Song , K. Yang , W. Zhao , L. Liu , J. Fang , G. Qian , F. Pan , L. Yang , Adv. Energy Sustainability Res. 2022, 3, 2200083.

[39] D. He , J. Lu , G. He , H. Chen , Front. Chem. 2022, 10, 916132. 35668827 10.3389/fchem.2022.916132 PMC9163830

[40] S. Tang , H. Zhao , RSC Adv. 2014, 4, 11251. 24729866 10.1039/C3RA47191H PMC3981120

[41] D. D. Lecce , V. Marangon , H. G. Jung , Y. Tominaga , S. Greenbaum , J. Hassoun , Green Chem. 2022, 24, 1021.

[42] Y. Zhou , T. Zhou , T. Ashirov , M. El Kazzi , C. Cancellieri , L. P. H. Jeurgens , J. W. Choi , A. Coskun , Nat. Commun 2022, 13, 2575. 35523785 10.1038/s41467-022-29199-3 PMC9076822

[43] V. Kumar , R. R. Reddy , B. V. N. P. Kumar , C. V. Avadhani , S. Ganapathy , N. Chandrakumar , S. Sivaram , J. Phys. Chem. C 2019, 123, 9661.

[44] Y. Yamada , A. Yamada , Chem. Lett. 2017, 46, 1056.

[45] Y. Yamada , A. Yamada , J. Electrochem. Soc. 2015, 162, A2406.

[46] V. L. Jr , C. Y. Kuo , C. W. Lan , ChemElectroChem 2023, 11, 202300501.

[47] P. E. Blöchl , Phys. Rev. B 1994, 50, 17953. 10.1103/physrevb.50.17953 9976227

[48] G. Kresse , J. Furthmüller , Phys. Rev. B 1996, 54, 11169. 10.1103/physrevb.54.11169 9984901

[49] G. Kresse , J. Furthmüller , Comput. Mater. Sci. 1996, 6, 15.

[50] J. P. Perdew , K. Burke , Y. Wang , Phys. Rev. B 1996, 54, 16533. 10.1103/physrevb.54.16533 9985776

[51] J. P. Perdew , K. Burke , M. Ernzerhof , Phys. Rev. Lett. 1996, 77, 3865. 10062328 10.1103/PhysRevLett.77.3865

[52] J. Heyd , G. E. Scuseria , M. Ernzerhof , J. Chem. Phys. 2003, 118, 8207.

[53] A. V. Krukau , O. A. Vydrov , A. F. Izmaylov , G. E. Scuseria , J. Chem. Phys. 2006, 125, 224106. 17176133 10.1063/1.2404663

