# Supplementary material for: Engineering of Aromatic Naphthalene and Solvent Molecules to Optimize Chemical Prelithiation for Lithium‐Ion Batteries
Source: Adv Sci (Weinh). 2024 Jun 18;11(30):2309155. doi: 10.1002/advs.202309155 (PMC11321689; doi:10.1002/advs.202309155)
Supplement: Supplementary file 1 — Supporting Information [file ADVS-11-2309155-s001.pdf]

ADVANCED SCIENCE
Open Access

Supporting Information

for *Adv. Sci.*, DOI 10.1002/advs.202309155

Engineering of Aromatic Naphthalene and Solvent Molecules to Optimize Chemical Prelithiation for Lithium-Ion Batteries

Jagabandhu Patra, Shi-Xian Lu, Jui-Cheng Kao, Bing-Ruei Yu, Yu-Ting Chen, Yu-Sheng Su, Tzi-Yi Wu, Dominic Bresser, Chien-Te Hsieh, Yu-Chieh Lo* and Jeng-Kuei Chang**

Copyright WILEY-VCH Verlag GmbH & Co. KGaA, 69469 Weinheim, Germany, 2023.

Supporting Information

Engineering of Aromatic Naphthalene and Solvent Molecules to Optimize Chemical Prelithiation for Lithium-Ion Batteries

Jagabandhu Patra#, Shi-Xian Lu#, Jui-Cheng Kao, Bing-Ruei Yu, Yu-Ting Chen, Yu-Sheng Su, Tzi-Yi Wu, Dominic Bresser, Chien-Te Hsieh, Yu-Chieh Lo*, Jeng-Kuei Chang**

Table S1. Summary of charge-discharge performance of pristine, Li-Naph/G1, Li-2-M-Naph/G1, and Li-1-M-Naph/G1 HC electrodes.

Specific current (mA g^{-1})	Pristine HC (mAh g^{-1})	Li-Naph/G1 (mAh g^{-1})	Li-2-M-Naph/G1 (mAh g^{-1})	Li-1-M-Naph/G1 (mAh g^{-1})
50	320	321	320	322
100	268	281	290	300
200	230	248	260	270
500	190	210	222	233
1000	165	178	189	200
2000	144	154	163	177
High rate retention (C_{50}/C_{2000})	45%	48%	51%	55%
Capacity retained after 500 cycles	80%	81%	84%	90%

Table S2. Summary of charge-discharge performance of Li-1-M-Naph/G1 Li-1-M-Naph/G2, and Li-1-M-Naph/G3 HC electrodes.

Specific current (mA g^{-1})	Li-1-M-Naph/G1 (mAh g^{-1})	Li-1-M-Naph/G2 (mAh g^{-1})	Li-1-M-Naph/G3 (mAh g^{-1})
50	322	320	321
100	300	269	258
200	270	230	210
500	233	178	156
1000	200	152	130
2000	177	125	109
High rate retention (C_{50}/C_{2000})	55%	39%	34%
Capacity retained after 500 cycles	90%	80%	78%

Table S3. Summary of charge-discharge performance of various HC electrodes prelithiated using Li-1-M-Naph/G1 solution with different aging times.

Specific current (mA g^{-1})	2 h (mAh g^{-1})	4 h (mAh g^{-1})	6 h (mAh g^{-1})	8 h (mAh g^{-1})	10 h (mAh g^{-1})	12 h (mAh g^{-1})
50	320	320	321	321	322	321
100	273	281	290	297	300	299
200	235	244	255	262	270	267
500	200	208	219	225	233	230
1000	170	178	184	194	200	199
2000	147	153	163	170	177	176
High rate retention (C_{50}/C_{2000})	46%	48%	51%	53%	55%	55%
Cycle retention after 500 cycles (%)	88%	91%	94%	96%	98%	98%

Table S4. Performance comparison of HC electrodes prelithiated using various approaches.

**Chemical prelithiation**								
Anode material	Solvents	Aromatic compounds	ICE	Prelithiation time	Low rate capacity	High rate capacity	Cycling stability	Ref.
Hard carbon	THF	Biphenyl	~106%	30 second	~288 Ah/g @30 mA/g	~50 Ah/g @1500 mA/g	stable up to 100 cycles @300 mA/g	1
Hard carbon	G1	Naphthalene	~99.5%	4 min	~400 mAh/g @50 mA/g	~195 mAh/g @4000 mA/g	stable up to 500 cycles @400 mA/g	2
Hard carbon	THF	4,4-dimethyl biphenyl	~100%	1 min	~275 mAh/g @15 mA/g	na	na	3
Hard carbon	THF	Biphenyl	96%	3 min	~270 mAh/g @37.2mA/g	~125 mAh/g @372mA/g	stable up to after 50 cycles @744 mA/g	4
**Hard carbon**	**G1**	**1-M-Naphthalene**	**98%**	**1 min**	**322 mAh/g @50 mA/g**	**~177 mAh/g @2000 mA/g**	**90% retention after 500 cycles @400 mA/g at 25 °C; 87% retention after 500 cycles @400 mA/g at 50 °C.**	**This work**
**Electrochemical prelithiation**								
Hard carbon	-	-	65%	-	250 mAh/g @50 mA/g	~100 mAh/g @1200 mA/g	stable up to 400 cycles @30 mA/g	5

Reference:

1. X. Zhang, H. Qu, W. Ji, D. Zheng, T. Ding, C. Abegglen, D. Qiu, D. Qu, *ACS Appl. Mater. Interfaces* **2020**, *12*, 11589.
2. Y. Shen, J. Qian, H. Yang, F. Zhong, X. Ai, *Small* **2020**, *16*, 1907602.
3. H. Yue, S. Zhang, T. Feng, C. Chen, H. Zhou, Z. Xu, M. Wu, *ACS Appl. Mater. Interfaces* **2021**, *13*, 45, 53996.
4. S. J. Hong, S. S. Kim, S. Nam, *Corros. Eng. Sci. Technol.* **2021**, *20, 15.*
5. M. Drews, J. Buttner, M. Bauer, J. Ahmed, R. Sahu, C. Scheu, S. Vierrath, A. Fischer, D. Biro, *ChemElectroChem* **2021**, *8*, 4750.

Table S5. Price comparison of various solvents and PAHs used for chemical prelithiation (according to https://www.tcichemicals.com).

**Solvents**	**Price (in USD)**
Tetrahydropyran	150/ 500 ml
2-methyl-Tetrahydrofuran	86/ 500 ml
ethylene glycol dimethyl ether	59/ 500 ml
diethylene glycol dimethyl ether	50/ 500 ml
triethylene glycol dimethyl ether	68/ 500 ml
**PAHs**	**Price (in USD)**
2-methylbiphenyl	50/1 gm
3,3',4,4'-Tetramethylbiphenyl	63.25/ 1 gm*
2-fluorobenzene	16/ 25 gm
4,4’-diethyl Biphenyl	119/ 25 gm
4-methylbiphenyl	262/ 25 gm
4,4′-di-tert-butylbiphenyl	88/ 25 gm
4,4′ -dimethylbiphenyl	150/ 25 gm
Cyano-naphthalene	68/ 25 mg
9,9- dimethyl-9H-fluorene	112/ 1 gm
Biphenyl	17/ 25 gm
Naphthalene	16/ 25 gm
2-methylnaphthalene	23/ 25 gm
1-methylnaphthalene	18/ 25 gm

* according to https://www.fishersci.com/us/en/home.html

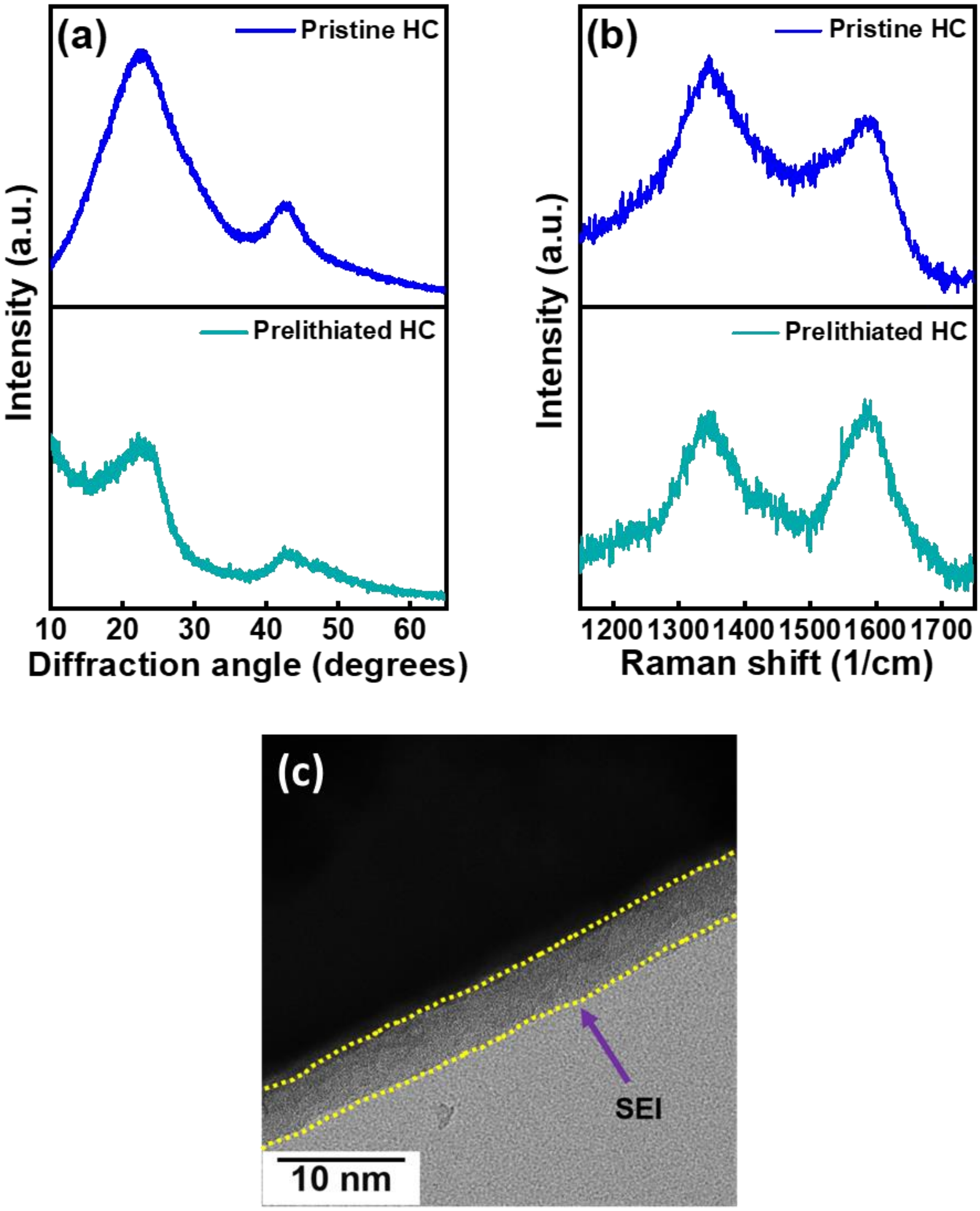

Figure S1. (a) X-ray diffraction patterns and (b) Raman spectra of pristine HC and HC prelithiated using Li-1-M-Naph/G1 solution. (c) TEM image of HC electrode prelithiated using Li-1-M-Naph/G1 solution.

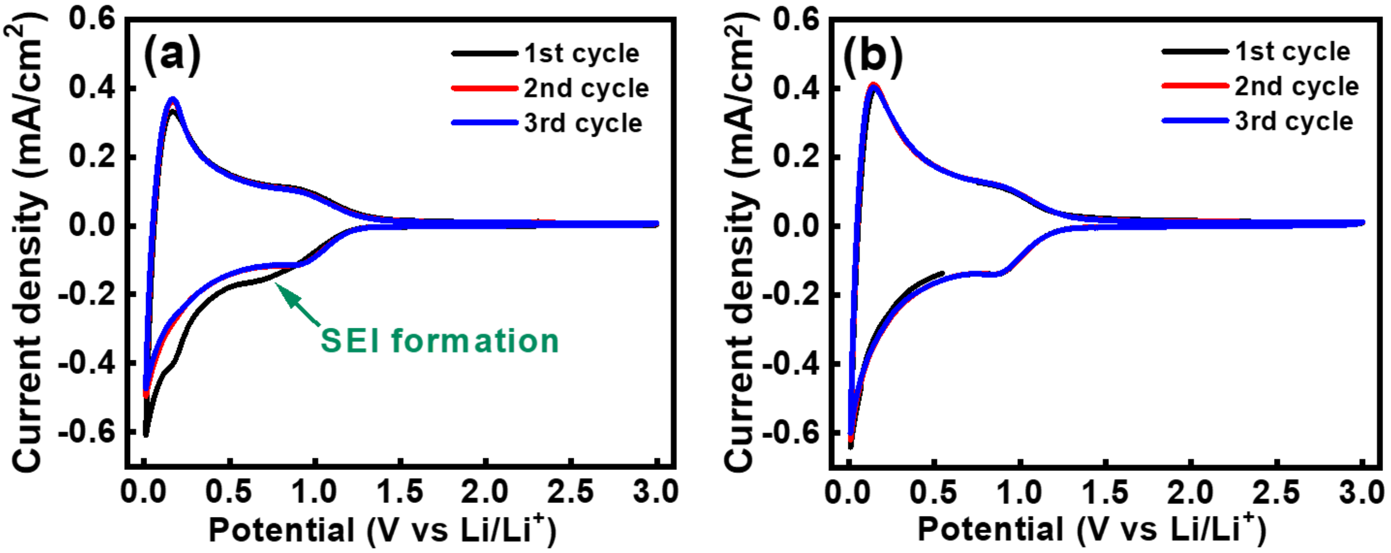

Figure S2. CV curves of (a) pristine and (b) Li-Naph/G1 HC electrode measured at potential scan rate of 0.2 mVs^{-1}.

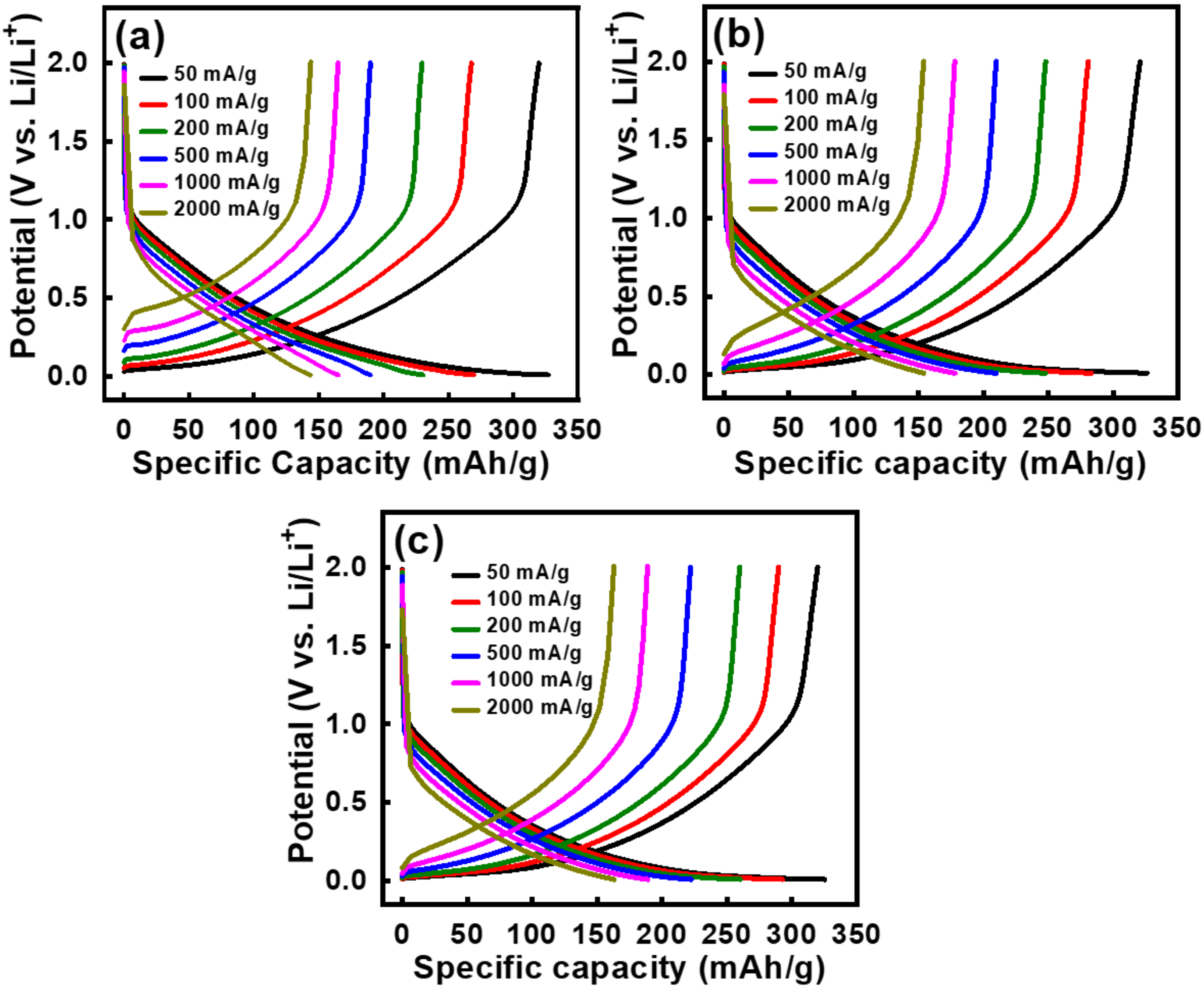

Figure S3. Charge-discharge curves of (a) pristine, (b) Li-Naph/G1, and (c) Li-2-M-Naph/G1 HC electrodes measured at various current rates.

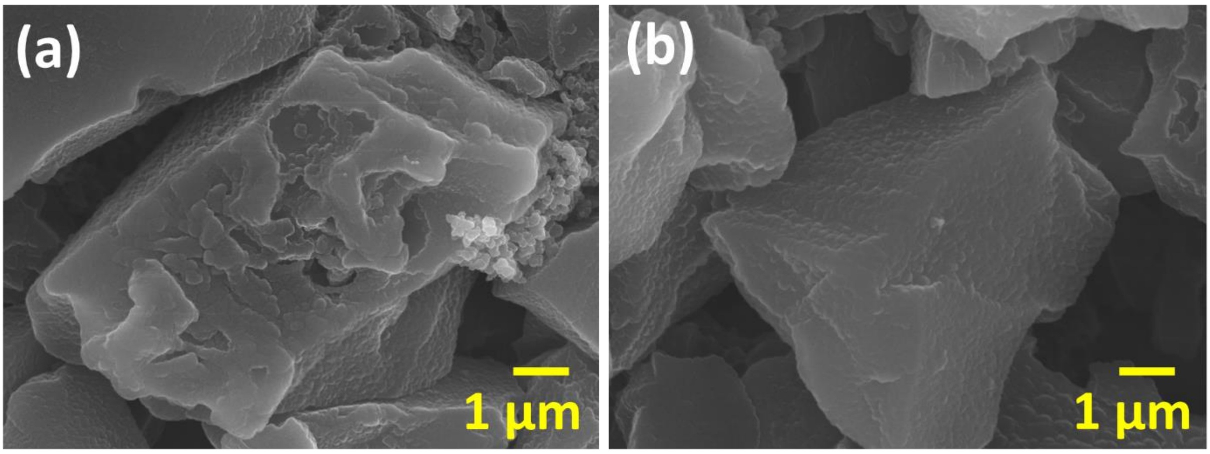

Figure S4. SEM images of (a) pristine and (b) Li-1-M-Naph/G1 HC electrodes after 500 charge-discharge cycles.

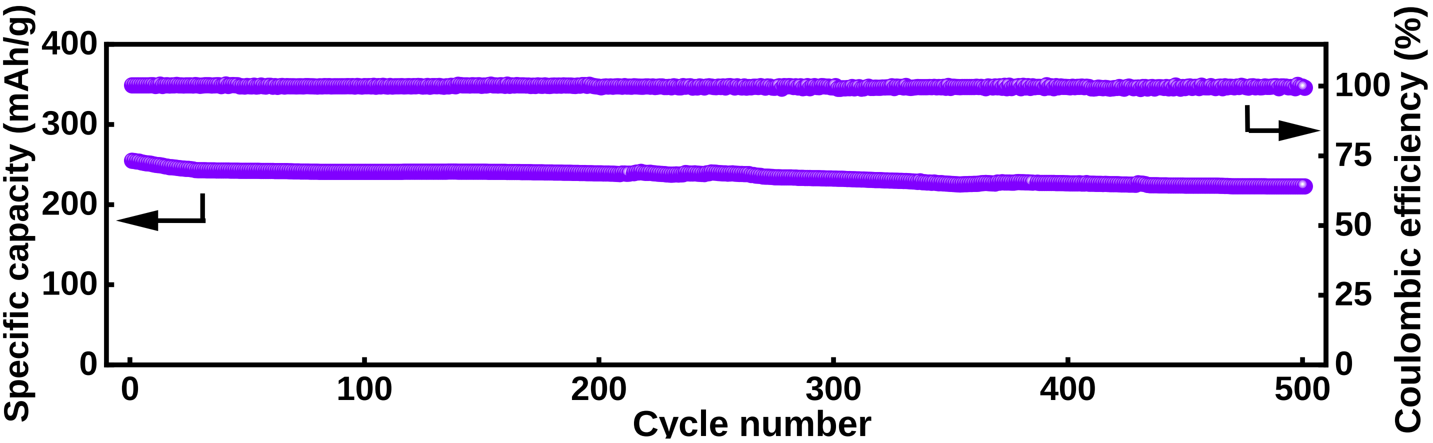

Figure S5. Cycling stability data of Li-1-M-Naph/G1 HC electrode measured at 400 mA g^{-1} for 500 cycles at 50 °C.

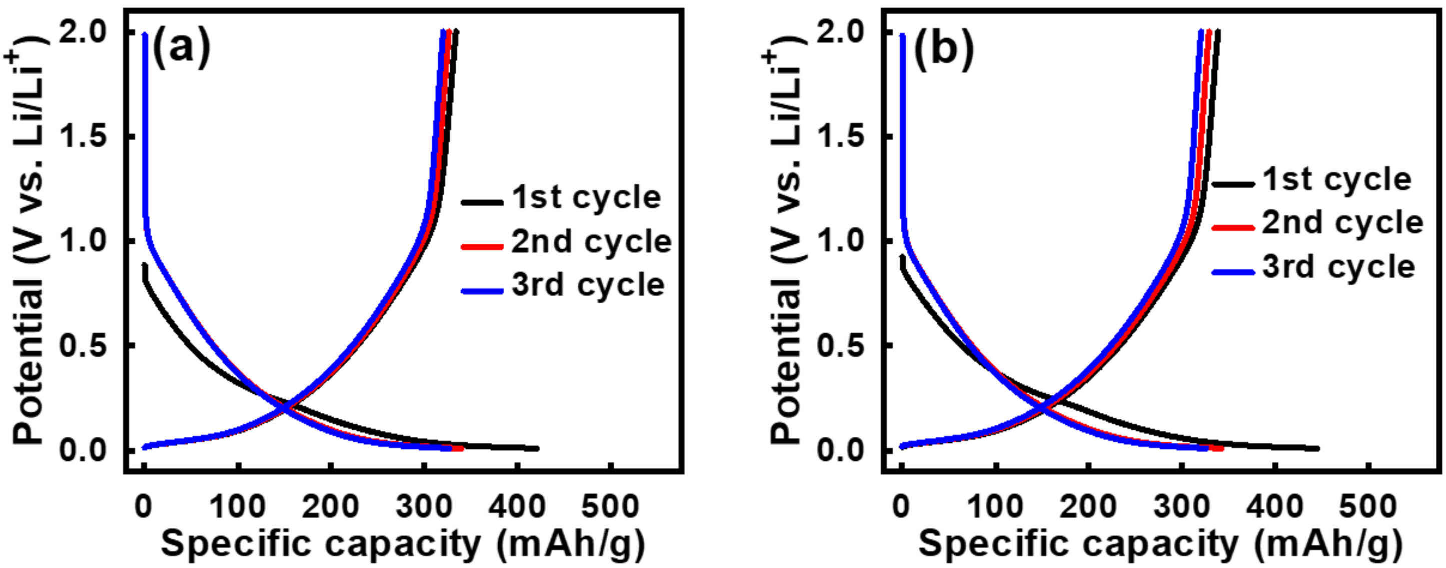

Figure S6. Initial charge-discharge curves of (a) Li-1-M-Naph/G2 and (b) Li-1-M-Naph/G3 HC electrodes measured at 50 mA g^{-1}.

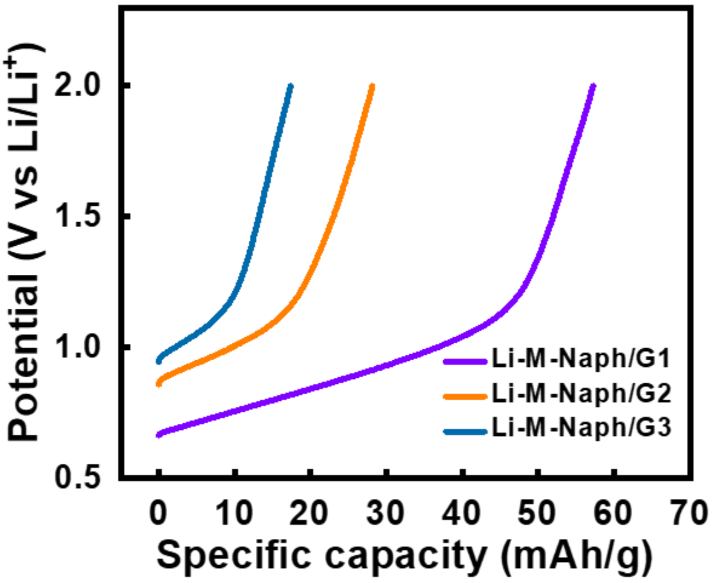

Figure S7. Direct-discharge curves of various prelithiated HC electrodes measured at 50 mA g^{-1}.

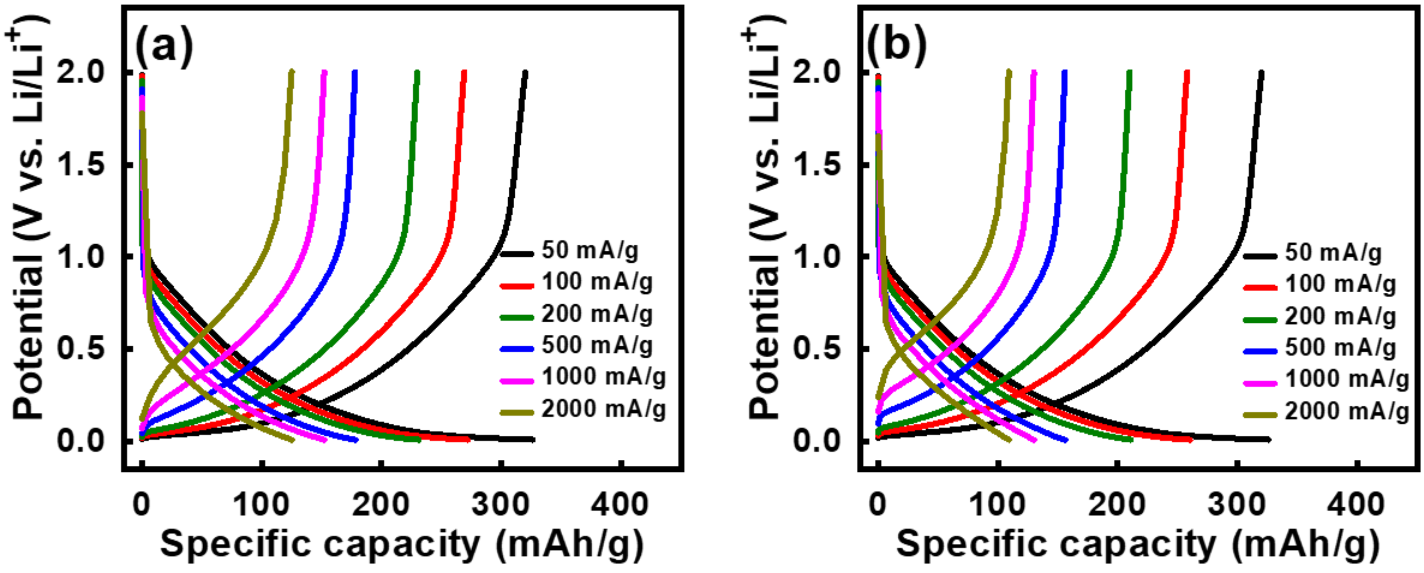

Figure S8. Charge-discharge curves of (a) Li-1-M-Naph/G2 HC and (b) Li-1-M-Naph/G3 HC electrodes measured at various current rates.

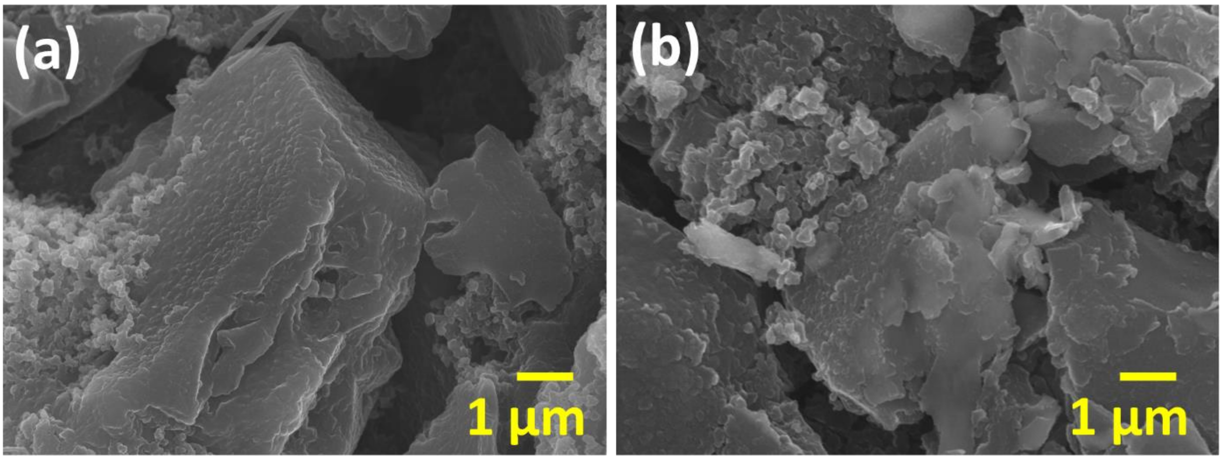

Figure S9. SEM images of (a) Li-1-M-Naph/G2 and (b) Li-1-M-Naph/G3 HC electrodes after 500 charge-discharge cycles.

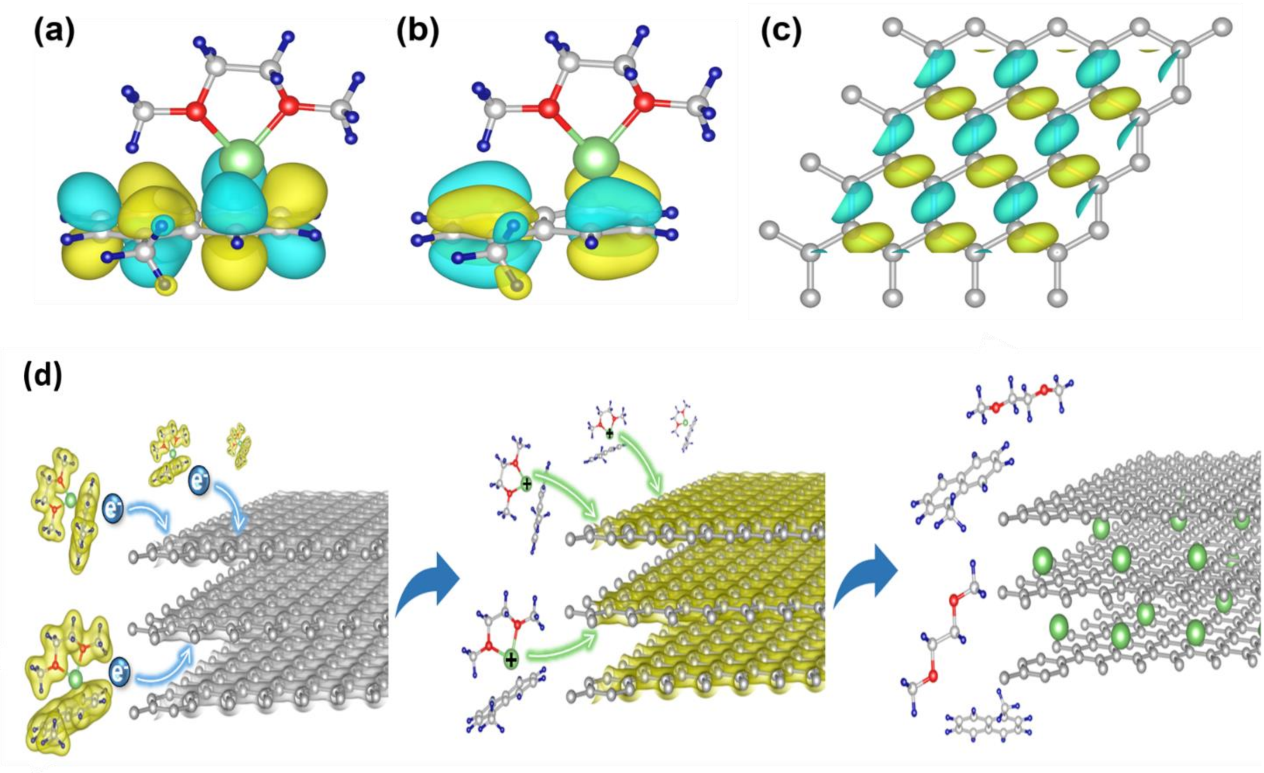

Figure S10. The wavefunction distribution of (a) α-HOMO of Li-1-M-Naph/G1, (b) β-HOMO of Li-1-M-Naph/G1, and (c) LUMO of graphene monolayer. (d) The proposed prelithiation mechanism. The yellow translucent area indicates the spatial distribution of electron charge within the molecules.

The lowest unoccupied molecular orbital (LUMO) energy levels for mono-layer, bi-layer, and tri-layer carbons are calculated to be −4.329, −4.360, and −4.351 eV, respectively (the vacuum level is aligned to 0 eV). By comparing these energy levels with those of the various Naph compounds (see **Figure 2(c)**), it is evident that the LUMO energy levels of the layered carbons are lower than the α-HOMO energy levels of LACs. Moreover, the wavefunction analysis shows a notable similarity in orbital symmetry between the frontier orbital of Li-1-M-Naph/G1 and a graphene layer, as shown in **Figure S10(a-c)**. Consequently, based on the frontier orbital theory,[1,2] electrons from the Naph compound can effectively transfer to the carbon layer. Subsequently, the negatively charged HC triggers the desolvation of Li^+ ions in the LAC solution and drives the Li^+ insertion into the carbon layers, as schemed in **Figure S10(d)**.

Reference

1. K. Fukui, T. Yonezawa, H. Shingu, J. Chem. Phys. 1952, 20, 722.
2. K. Fukui, T. Yonezawa, C. Nagata, H. Shingu, J. Chem. Phys. 1954, 22, 1433.

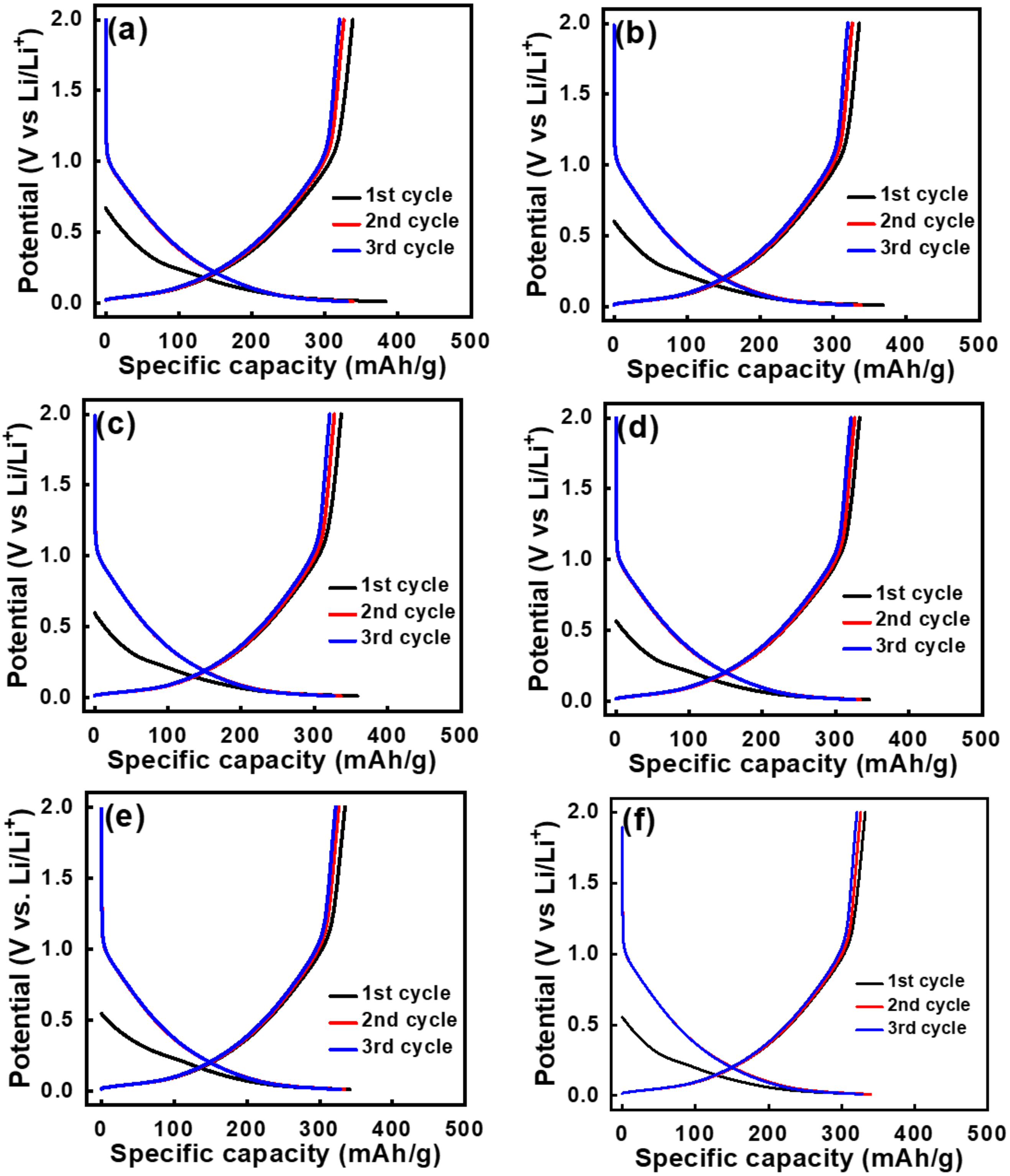

Figure S11. Initial charge-discharge curves (measured at 50 mA g^{-1}) of HC electrodes prelithiated using Li-1-M-Naph/G1 solution with aging times of (a) 2 h, (b) 4 h, (c) 6 h, (d) 8 h, (e) 10 h, and (f) 12 h.

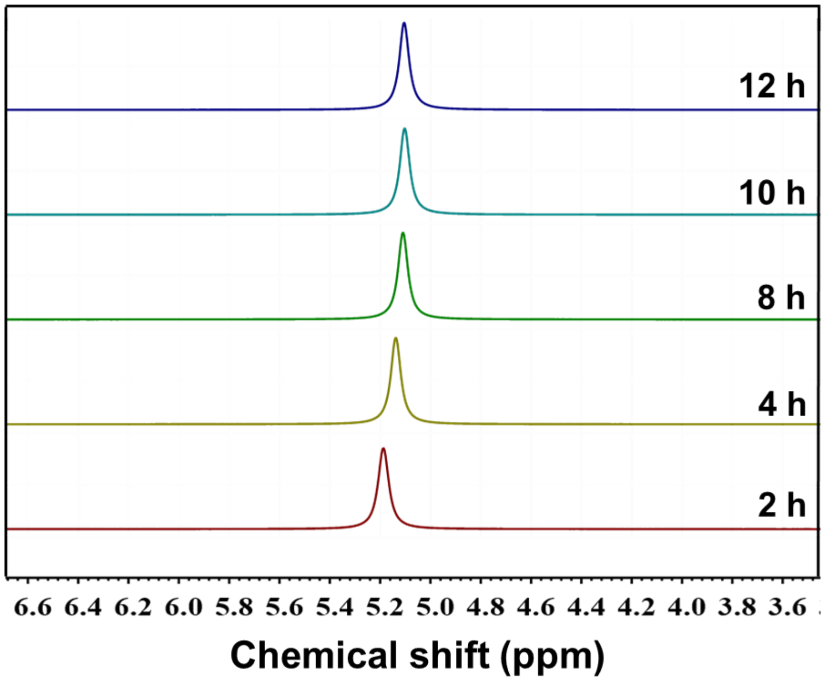

Figure S12. ^{7}Li NMR spectra of Li-1-M-Naph/G1 solution with various aging times.

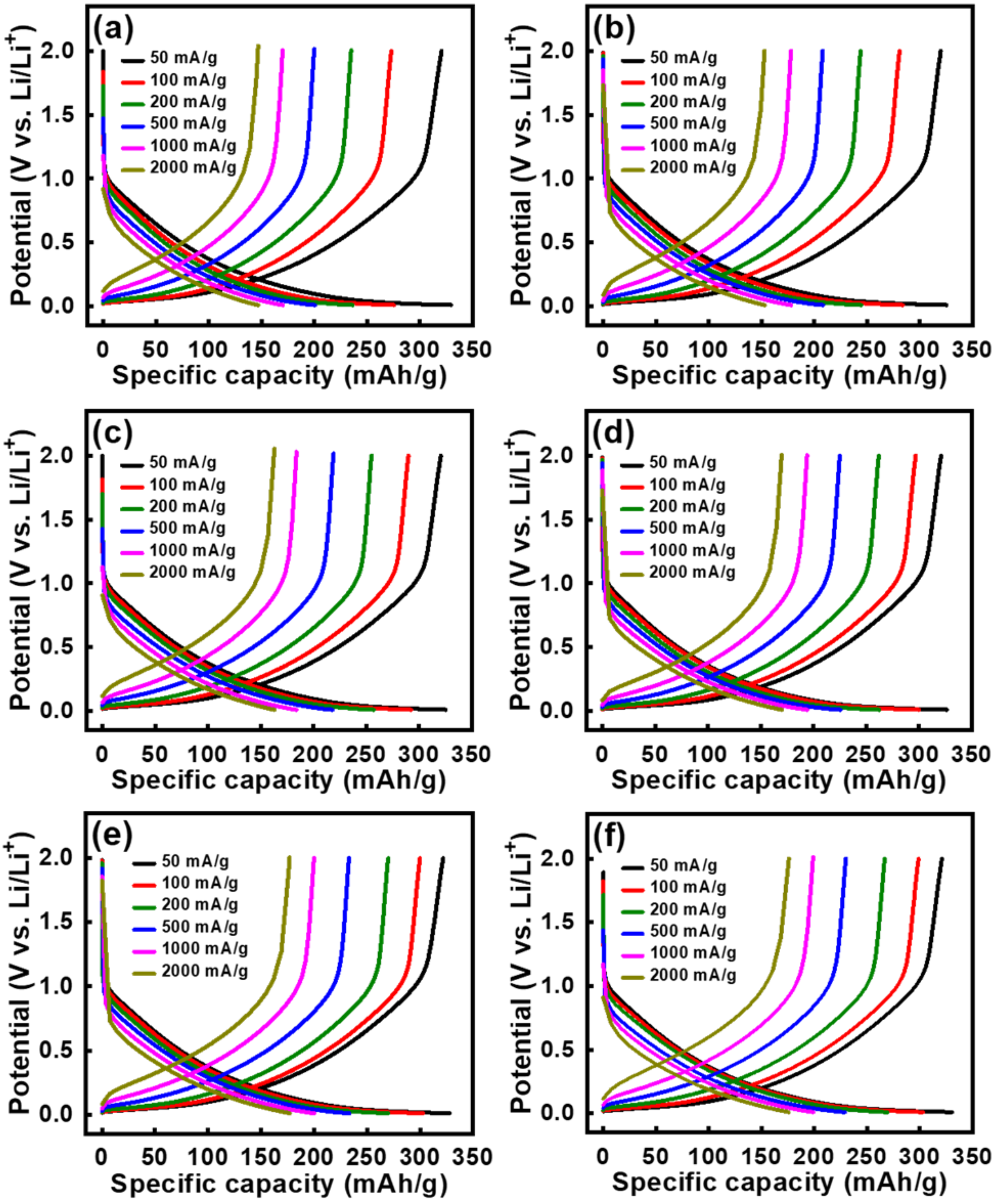

Figure S13. Charge-discharge curves (measured at various current rates) of HC electrodes prelithiated using Li-1-M-Naph/G1 solution with aging times of (a) 2 h, (b) 4 h, (c) 6 h, (d) 8 h, (e) 10 h, and (f) 12 h.

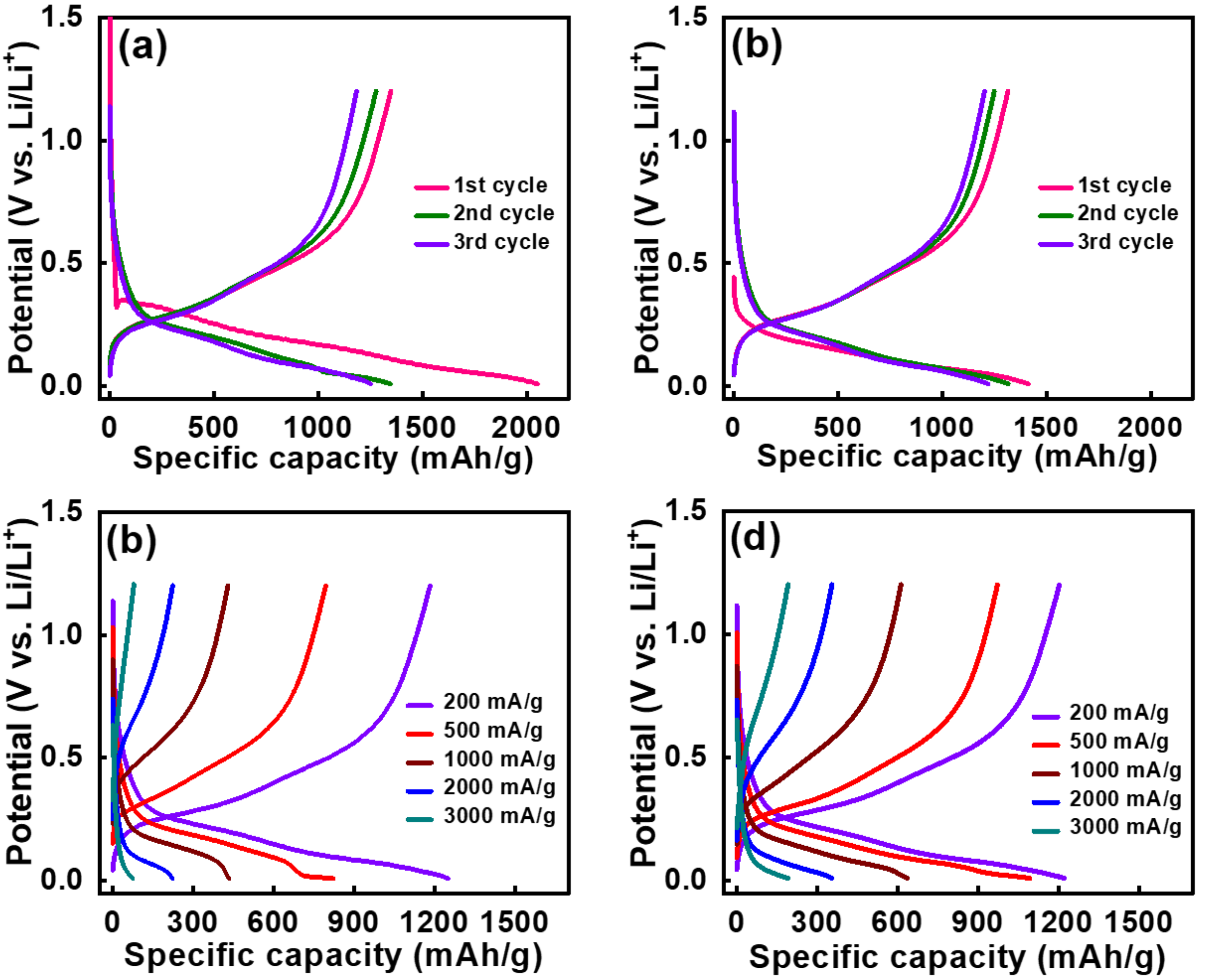

Figure S14. Initial charge-discharge curves measured at 200 mA g^{-1} for (a) pristine SiO_x and (b) prelithiated SiO_x electrodes. Charge-discharge profiles measured at various rates for (c) pristine SiO_x and (d) prelithiated SiO_x electrodes. The Li-1-M-Naph/G1 solution was used for prelithiation.

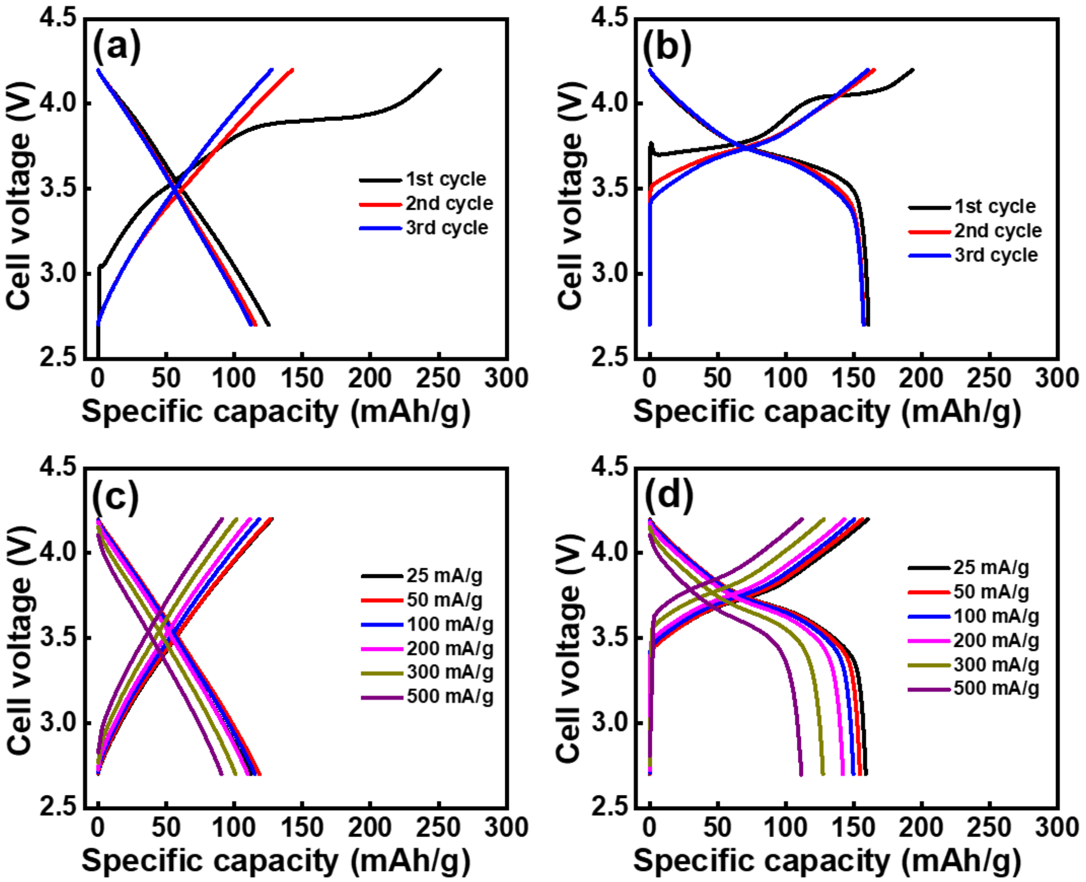

Figure S15. Initial charge-discharge curves measured at 25 mA g^{-1} (based on $LiNi_{0.6}Co_{0.2}Mn_{0.2}O_2$) for (a) pristine HC||$LiNi_{0.6}Co_{0.2}Mn_{0.2}O_2$ and (b) prelithiated HC||$LiNi_{0.6}Co_{0.2}Mn_{0.2}O_2$ full cells. Charge-discharge profiles measured at various rates for (c) pristine HC||$LiNi_{0.6}Co_{0.2}Mn_{0.2}O_2$ and (d) prelithiated HC||$LiNi_{0.6}Co_{0.2}Mn_{0.2}O_2$ full cells. The Li-1-M-Naph/G1 solution was used for prelithiation.
